# Comprehensive review of machine learning and deep learning techniques for epileptic seizure detection and prediction based on neuroimaging modalities

**DOI:** 10.1186/s42492-025-00208-8

**Published:** 2025-12-11

**Authors:** Khadija Slama, Ali Yahyaouy, Jamal Riffi, Mohamed Adnane Mahraz, Hamid Tairi

**Affiliations:** Laboratory of Computer Science, Innovation, and Artificial Intelligence, Department of Computer Science, Faculty of Sciences Dhar El Mahraz, Sidi Mohamed Ben Abdellah University, Fez, 30000 Morocco

**Keywords:** Epileptic seizures, Electroencephalogram, Feature extraction, Machine learning, Deep learning

## Abstract

Epilepsy is a chronic neurological disorder characterized by recurrent seizures that can lead to death. Seizure treatment usually involves antiepileptic drugs and sometimes surgery, but patients with drug-resistant epilepsy often remain effectively untreated owing to the lack of targeted therapies. The development of a reliable technique for detecting and predicting epileptic seizures could significantly impact clinical treatment protocols and the care of patients with epilepsy. Over the years, researchers have developed various computational techniques using scalp electroencephalography (EEG), intracranial EEG, and other neuroimaging modalities, evolving from traditional signal processing methods (e.g., wavelet transforms and template matching) to advanced machine learning (ML, e.g., support vector machines and random forests) and deep learning (DL) algorithms (e.g., convolutional neural networks, recurrent neural networks, transformers, graph neural networks, and hybrid architectures). This review provides a detailed examination of epileptic seizure detection and prediction, covering the key aspects of signal processing, ML algorithms, and DL techniques applied to brainwave signals. We systematically categorized the techniques, analyzed key research trends, and identified critical challenges (e.g., data scarcity, model generalizability, and real-time processing). By highlighting the gaps in the literature, this review serves as a valuable resource for researchers and offers insights into future directions for improving the accuracy, interpretability, and clinical applicability of EEG-based seizure detection systems.

## Introduction

Epilepsy is a neurological disorder characterized by recurrent, unprovoked seizures, which are sudden and temporary perturbations of the brain’s normal electrical activity. It affects millions of people worldwide and imposes significant burdens on individuals and healthcare systems. According to the World Health Organization, more than 50 million people worldwide suffer from epilepsy, and nearly 80% of them live in low- and middle-income countries [1].

Individuals with epilepsy experience unexpected and abrupt seizures that can cause them to lose consciousness and become uncontrollable. Hence, they are susceptible to injuries, traffic accidents, and death. These seizures vary widely in severity, duration, and symptoms, ranging from lapsed awareness to convulsions and unconsciousness.

**Fig. 1:**
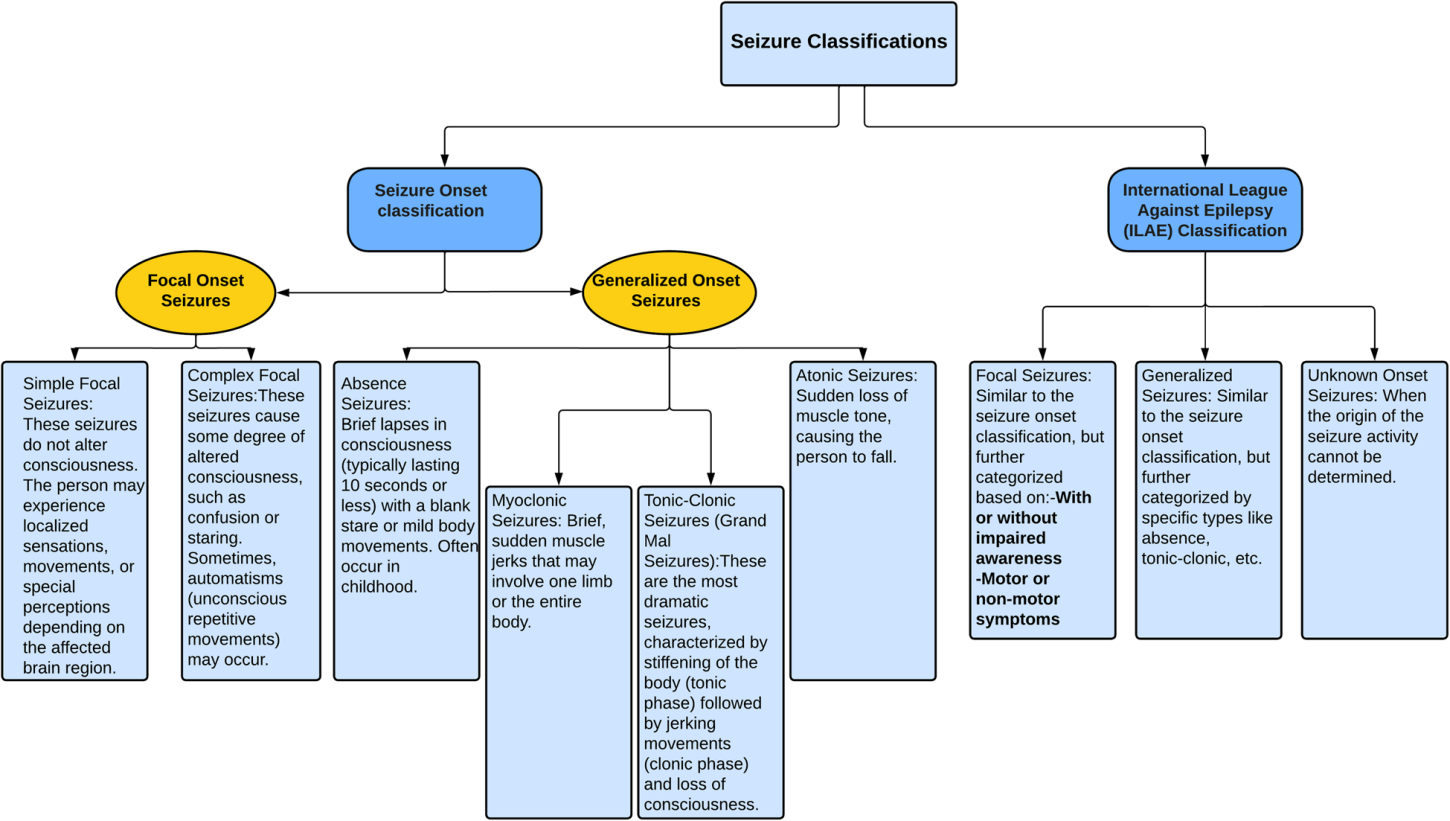
Classification of epilepsy by seizure onset and using the International League Against Epilepsy classification

As Fig. 1 shows, epilepsy encompasses a range of seizure disorders, including generalized epilepsy [2, 3], which affects consciousness and involves both hemispheres of the brain, and partial (focal) epilepsy [4, 5], where seizures originate in a specific brain region, potentially impairing consciousness or not. Unknown-onset epilepsy [6] refers to the type of epilepsy where the exact onset or cause of the seizures is not easily identifiable or understood. In such cases, there is uncertainty regarding whether the seizures originate from a specific brain region (focal onset) or simultaneously affect both hemispheres (generalized onset). General epilepsy affects the entire brain and disrupts all neuronal activity, eventually including the whole brain [7–9].

The latest research revealed that, with proper diagnosis and treatment, 70% of epilepsy patients could achieve a seizure-free life [1]. However, identifying this condition early and accurately is critical. Artificial intelligence (AI) plays a crucial role in this area by enabling the automated analysis and interpretation of large-scale and complex biomedical signals.

Various neuroimaging techniques [10] have been employed to develop accurate solutions for detecting epilepsy. Among these techniques are structural and functional neuroimaging tools such as magnetoencephalography (MEG) [11, 12], functional magnetic resonance imaging (fMRI) [13, 14], and positron emission tomography (PET) [15, 16], which excel at localizing epileptogenic zones using anatomical and metabolic biomarkers.

However, electroencephalography (EEG) [17, 18] has emerged as the dominant modality for diagnosis due to its high temporal resolution in capturing ictal and interictal electrophysiological patterns, despite its limited spatial resolution. Most studies on epilepsy detection and prediction have focused on developing automated solutions, which rely on EEG signals as the primary modality given their high temporal resolution, direct measurement of neuronal activity, and widespread clinical use in seizure analysis. Other neuroimaging techniques, i.e., PET and MRI, provide valuable structural and metabolic insights; however, their application in AI-based epilepsy research remains limited owing to several factors. PET and MRI offer superior spatial resolution but lack the millisecond-level precision needed to capture rapid epileptiform discharges, making them less suitable for real-time seizure detection and prediction. In addition, EEG is more accessible, cost-effective, and easier to integrate into continuous monitoring systems than PET and MRI, which require specialized facilities and are less practical for long-term use. Despite their potential for localizing epileptogenic zones and studying metabolic abnormalities, the high computational demands and smaller annotated datasets for PET and MRI have hindered their widespread adoption in AI models. Therefore, current research remains predominantly focused on EEG modalities.

By focusing on the historical evolution of automated seizure detection and prediction techniques, we can trace their origins back to pioneering efforts, starting with Gotman [19], who first proposed a method for automatically detecting epileptic episodes. Further developments allowed Qu and Gotman [20] to enhance the method by incorporating longer temporal characteristics and utilizing the commonalities in neural oscillations. Park et al. [21] suggested a model for detecting seizures in long-term brainwave signals associated with epilepsy by utilizing an artificial neural network (ANN) combined with the wavelet transform (WT) technique. Subasi and Erçelebi [22] utilized neural networks with logistic regression (LR) to categorize brain wave signals as epileptic or normal. In 2008, Meier et al. [23] presented a new method for the real-time detection of different ictal events in human brainwave recordings, demonstrating its efficiency and promise for early seizure detection. Similarly, Khalil et al. [24] proposed two efficient frameworks based on statistical analysis: one for seizure detection using scale-invariant feature transform applied to 2D EEG representations, and another for seizure prediction using probabilistic thresholds derived from wavelet-based features. Over time, an increasing number of machine learning (ML) techniques have emerged that employ diverse methods to improve the efficiency and reliability of seizure identification. These methods require manual feature extraction using techniques that combine frequency, time, and time-frequency approaches [25–29]. However, the required manual parameter tuning results in time-intensive procedures that rely significantly on practical knowledge. Several challenges are associated with using conventional methods, such as selecting the most meaningful channels, determining appropriate decomposition stages in techniques such as discrete wavelet transform (DWT) [30, 31] or tunable-Q factor WT [32–34], and identifying a sufficient number of intrinsic modes in empirical mode decomposition (EMD) and variational mode decomposition techniques. Moreover, the choice of the most suitable and accurate ML algorithms, such as support vector machines (SVMs), random forests (RFs), and decision trees, requires testing and refinement. Typically, the selection of features and classifiers in conventional ML requires an iterative experimental process to achieve optimal results.

Although conventional ML techniques present specific advantages, it is important to understand their limitations. These methodologies often require specialized knowledge and manual adjustment to optimize parameters, which makes them labor-intensive and prone to bias. Given these factors, the development of deep learning (DL) methods offers a promising avenue for overcoming the challenges presented by conventional techniques. In 2014, Turner et al. [35] used deep belief networks to identify seizure events using multi-channel EEG signals. In 2016, Vidyaratne et al. [36] introduced an innovative deep bidirectional recurrent neural network (RNN) for processing brainwave signals to enhance seizure detection. In 2017, Talathi [37] used a gated recurrent unit (GRU) on the University of Bonn (Bonn) dataset and achieved an accuracy score of 100%. Golmohammadi et al. [38] suggested a novel technique featuring a hybrid architecture combining a convolutional neural network (CNN) and a long short-term memory (LSTM) component for epileptic signal classification. In the following year, Abdelhameed et al. [39] developed an automatic seizure identification method using deep convolutional autoencoders (AEs). These studies demonstrated that DL models improved the performance of EEG-based epileptic seizure detection and prediction compared with traditional ML methods. This improvement results from the ability of DL architectures to extract intricate and discriminative patterns.

This review systematically examines feature extraction techniques and their pivotal roles in seizure detection systems, encompassing both traditional ML and DL approaches. First, we explore conventional feature engineering methods and highlight their strengths and limitations in biomedical signal processing. Subsequently, we analyze how DL architectures automate feature extraction and offer comparative advantages in handling complex neurophysiological data. This study further evaluated state-of-the-art ML/DL hybrid models for their clinical applicability, providing critical insights into methodological trends and performance benchmarks. Finally, we discuss the open challenges and future directions for optimizing automated seizure detection frameworks.

In contrast to prior review papers in this field that primarily focused on systematic examinations of ML and DL applications, our study adopted a more inclusive methodology, systematically classifying and analyzing a diverse range of existing state-of-the-art approaches. Accordingly, the key contributions of this review are outlined below:

- A detailed overview of the preprocessing and feature extraction techniques, along with comparisons of the most commonly employed methods for EEG-based epilepsy detection and prediction.
- A summary of studies utilizing neuroimaging modalities other than EEG signals.
- Analysis of ML-based studies, including a comparison of algorithms used across different approaches.
- An investigation of DL models encompassing both single-architecture approaches (e.g., CNN and RNN) and hybrid models (e.g., CNN and CNN-transformer).
- An examination of the current limitations and future directions, addressing ongoing challenges, and identifying promising opportunities for advancing EEG-based seizure detection and prediction through DL.

This article is structured as follows: BCI section introduces the basic concepts of brain-computer interfaces (BCIs). EEG section discusses EEG as a BCI modality. Datasets and preprocessing methodology section provides publicly available datasets for EEG-based research using preprocessing methods. Feature extraction and selection section explores feature extraction techniques used to prepare the data for analysis. Epileptic seizure detection and prediction approaches section examines ML and DL methods employed to identify epileptic seizures. Finally, the key takeaways and potential future directions in this field is summarized.

## BCI

BCI technology represents a revolutionary leap in neuroscience and human-computer interaction by establishing a direct communication link between the brain and an external device. BCIs are extensively utilized for various applications in the medical and engineering fields.

**Fig. 2:**
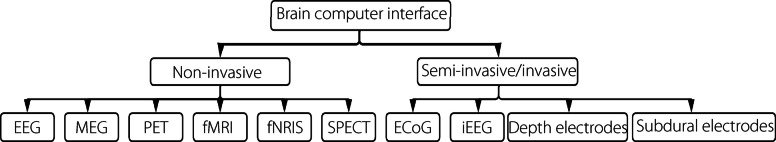
Neuroimaging techniques for detecting epileptic seizures

Neuroimaging techniques are indispensable for capturing brain activity and exploring neurological disorders. These techniques involve non-invasive, semi-invasive, and invasive methods for obtaining brain signals. The invasive approaches implant the microelectrodes directly into the cerebral cortex. Meanwhile, the semi-invasive methods entail placing electrodes on the brain surface to record electrical signals originating from the arachnoid and dura. Non-invasive techniques utilize scalp electrodes to gauge the electrical currents generated by the brain. Despite the high signal quality attained by invasive BCIs, the associated neurosurgery for electrode implantation poses considerable risks and costs. Electrocorticography, which requires craniotomy for electrode placement, is reserved for situations requiring surgical intervention. Nonetheless, it offers superior spatial resolution and immunity to artifacts and noise. Non-invasive methods can be classified into metabolic-based techniques, such as functional near-infrared spectroscopy, fMRI, and PET, and electrophysiological approaches, such as EEG and MEG, each offering distinct advantages for studying brain function and pathology [40]. Figure 2 lists various neuroimaging techniques.

Significant research efforts are currently underway in the field of detecting and predicting epileptic seizures using brain signals captured by EEGs [41, 42]. As previously noted, epileptic seizures are of various types, and the precise identification of seizure categories utilizing neurophysiological methods like EEG signals is crucial for neurologists [41, 42]. Moreover, physicians require the accurate detection of epileptic seizure types to prescribe appropriate medication [41, 42]. Additionally, detection helps in preventing drug-resistant epileptic seizures in patients [42].

## EEG

**Fig. 3:**
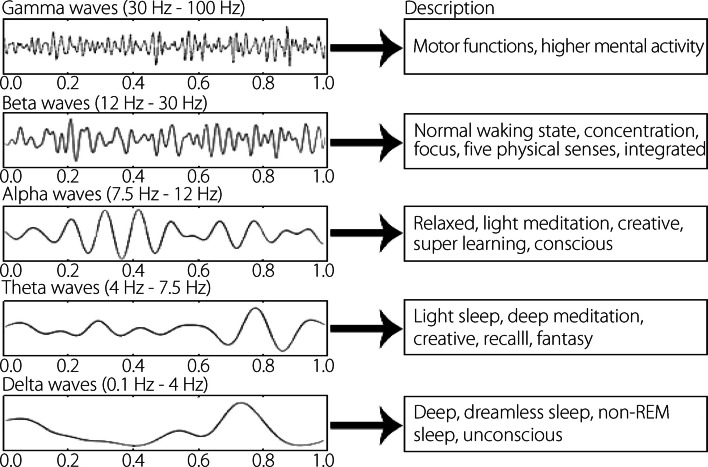
Brain wave descriptions

EEG is a commonly used non-invasive technique for monitoring electrical signals in the brain. It involves placing electrodes on the scalp to detect and record spontaneous electrical impulses generated by neurons. EEG is widely used in clinical settings to diagnose neurological disorders, such as epilepsy, stroke, and Alzheimer’s disease. The EEG signals typically display rhythmic oscillations known as brainwaves, which are categorized into different frequency ranges: alpha, beta, gamma, theta, and delta (Fig. 3). Each frequency band is associated with a specific brain state and function. For example, delta waves occur during deep sleep, theta waves occur during meditation or drowsiness, alpha waves occur during relaxed wakefulness, beta waves occur during active concentration, and gamma waves occur during cognitive processing. The amplitudes and frequencies of these oscillations provide valuable insights into brain function and dysfunction, aiding in the diagnosis and monitoring of neurological diseases. EEGs are also valuable in neuroscience research, enhancing the ability to understand brain dynamics, cognitive processes, and how neurological disorders affect brain function. Despite limitations, such as susceptibility to noise and artifacts, EEG remains widely used in both research and clinical practice because it is noninvasive, relatively low-cost, portable, and can provide real-time information.

### EEG electrodes

EEG electrodes are essential components of EEG, enabling the measurement and recording of electrical activity in the brain. Typically fabricated from conductive materials, such as metals, these electrodes are strategically placed on the scalp according to a standardized pattern, such as the International 10–20 system. Each electrode is connected to an amplifier that detects and amplifies the electrical impulses generated by neuronal activity in the brain. Electrodes are typically of the wet or dry types. Wet electrodes, often made of silver/silver chloride, require an electrolyte gel to reduce impedance at the skin-electrode interface. This reduction is critical for capturing high-quality EEG signals. In contrast, dry electrodes eliminate the gel, offering a more convenient but sometimes less precise alternative.

**Fig. 4:**
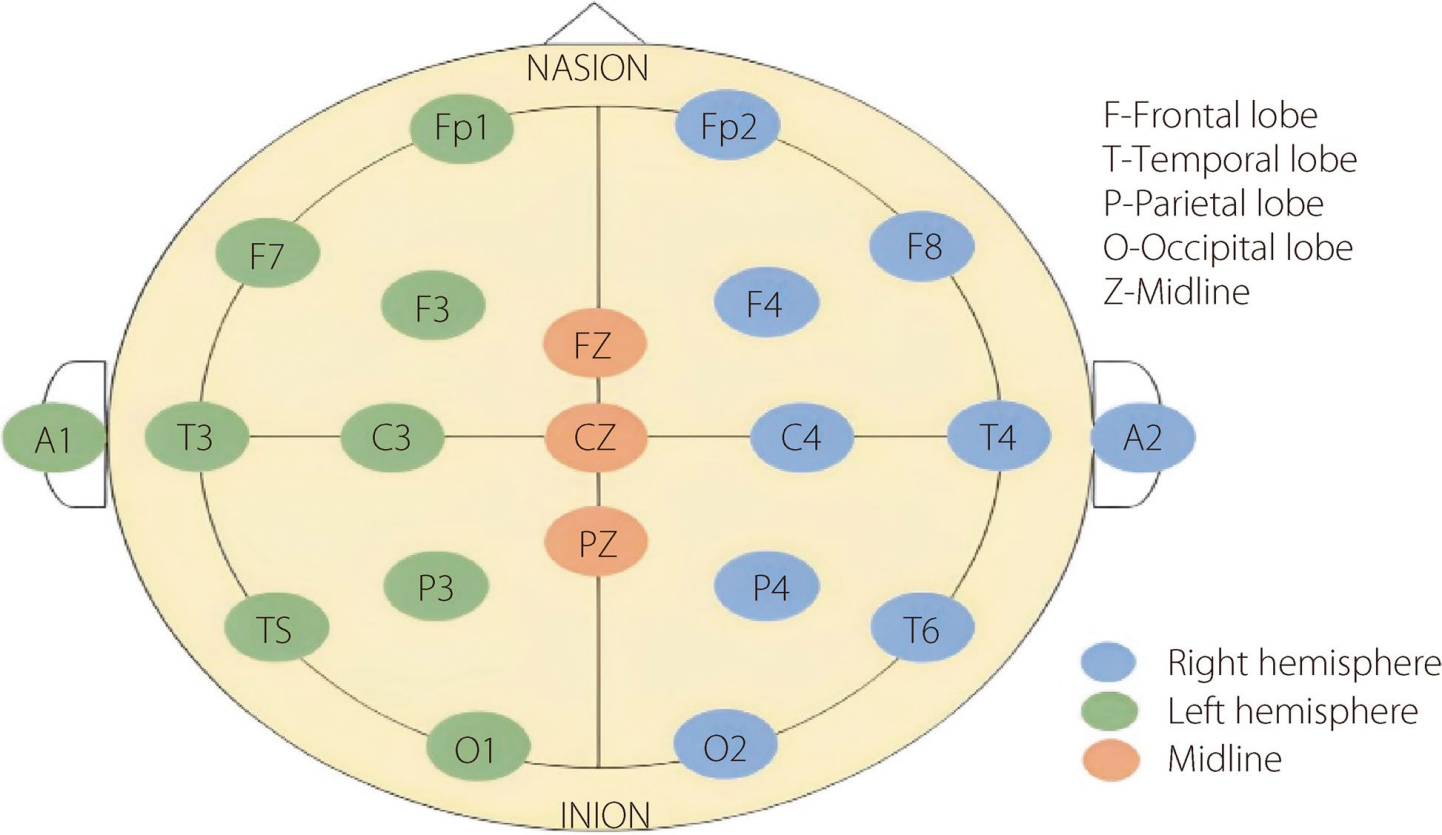
10–20 International EEG electrode placement system

Both types have their advantages, catering to different requirements in clinical and research environments. The proper positioning of EEG electrodes is essential for obtaining meaningful brain activity data. Following the International 10–20 system guidelines, electrodes are positioned according to anatomical landmarks on the scalp and segmented by percentages of the distances between these points. This system, endorsed by the International Federation of Clinical Neurophysiology and International League Against Epilepsy, correlates electrode placement with specific areas of the cortical surface (Fig. 4). The system adapts to the size and shape of the skull, ensuring that all brain areas are covered while maintaining consistent spacing between electrodes. Each location is designated by a letter and a number: letters indicate the lobe (P for parietal, T for temporal, F for frontal, C for central, and O for occipital), and a number or the letter ‘Z’ indicates the cerebral hemisphere or midline. Proper electrode placement is paramount for accurately localizing brain activity and minimizing artifacts that can arise from poor positioning or impedance issues. Consistent with this, correct placement ensures the reliability of EEG recordings, a critical factor in research and clinical applications.

### EEG disturbances

**Fig. 5:**
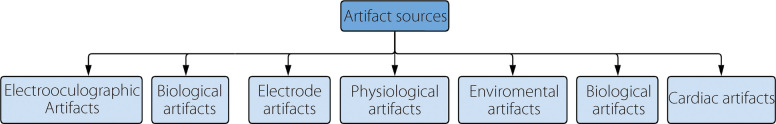
EEG signal artifact sources

During EEG recordings, undesired electrical activity and distortions, collectively termed EEG noise and artifacts, often compete with brain signals. The sources of artifacts in the EEG recordings are shown in Fig. 5. These can significantly hinder the interpretation of the EEG signals and obscure important information.

EEG noise encompasses inherent electrical activity originating outside the brain and is further differentiated as being physiological and environmental. Physiological noise arises in the body. Muscular activity, particularly of the scalp and jaw muscles, can mimic brain activity through electrical signals. Even subtle movements, such as blinking or sweating, can introduce noise. Conversely, environmental noise disrupts the EEG through electrical influences. Interference from nearby electrical equipment and environmental electromagnetic fields can contaminate the delicate brain signals.

EEG artifacts are not physiological but arise because of technical issues or patient movement. Electrode artifacts affect recordings when electrodes loosen or poorly contact the scalp, introducing spurious electrical signals. Motion artifacts pose a significant challenge because head movements, coughing, and swallowing can generate large electrical disturbances that overwhelm brain signals.

Minimizing the EEG noise and artifacts is paramount for obtaining clean and interpretable EEG recordings, and several techniques have been developed to overcome these challenges. Proper electrode placement ensures good contact with the scalp, while the conductive gel improves signal quality by facilitating electrical transmissions, with unwanted frequencies being removed from the raw EEG data by filtering. More complex cases may require advanced signal processing methods to isolate the brain’s subtle electrical signals from noise and artifact interference. Mitigating such disturbances in EEG recordings provides a clearer window into the intricacies of brain functions, aiding the identification of various neurological pathologies and supporting therapeutic interventions.

## Datasets and preprocessing methodology

### Publicly available datasets

**Fig. 6:**
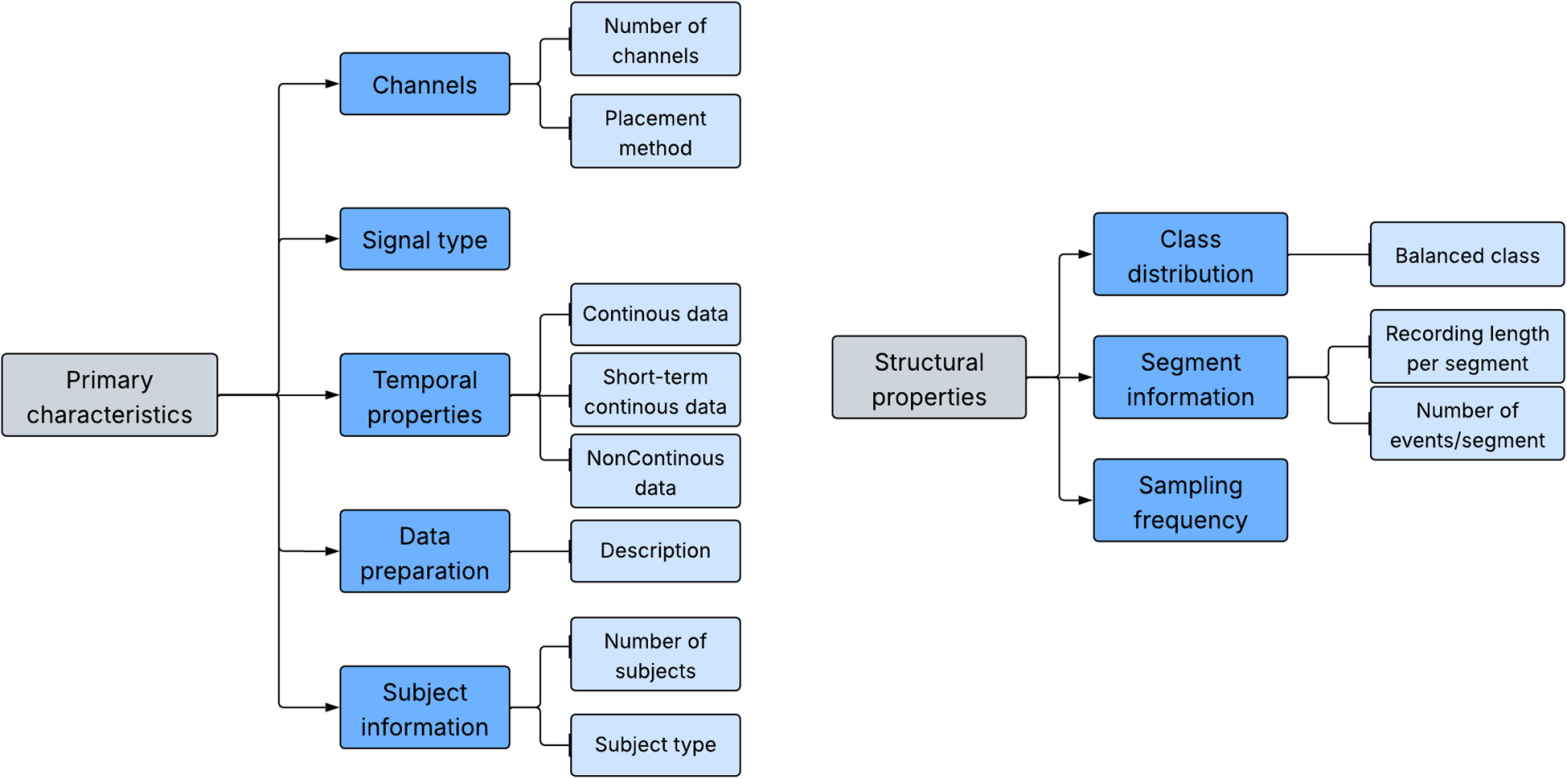
Fundamental characteristics and structural attributes of the available datasets

Publicly available datasets for epilepsy research are essential for advancing our understanding of the condition and facilitating the development of novel diagnostic and treatment strategies. They cover a broad spectrum of information, including clinical data, neuroimaging scans, and electrophysiological recordings collected from individuals with epilepsy. Multiple EEG datasets, namely Children’s Hospital Boston-Massachusetts Institute of Technology (CHB-MIT) [43], Freiburg [44], Bonn [45], Kaggle [46], Hauz Khas [47], Zenodo [48], Flint Hills [49], and Bern-Barcelona [50], are accessible for developing automated systems designed to detect and predict epileptic seizures. Their availability enables researchers worldwide to access standardized, high-quality data, foster collaboration, and accelerate scientific discovery. Furthermore, these datasets promote transparency and reproducibility by validating scholarly and methodological findings. By leveraging them, researchers can address key research questions, develop predictive models, and ultimately improve clinical outcomes for individuals living with epilepsy. Table 1 presents more information regarding publicly available datasets according to their primary characteristics and structural properties, as shown in Fig. 6.

**Table 1:** List of publicly available datasets

Dataset	Number of patient	Number of channel	Placement method	Type of signal	Number of event/segment	Sampling frequency	Balanced class
University of Bonn	10	1	International 10–20 system, intracranial	Scalp/intracranial EEG	500 segments	173.61	Yes
CHB-MIT	22	23	International 10–20 system/nomenclature	Scalp EEG	198 events	256	No
TUH EEG Seizure Corpus	642	24–36	International 10–20 system/nomenclature	Scalp EEG	3050 events	Minimum of 250	No
Melbourne- NeuroVista seizure trial (NeuroVista Ictal)	12	16	Intracranial	Intracranial EEG	2979 segments	400	No
Neurology and Sleep Centre Hauz Khas	10	19	International 10–20 system	Scalp EEG	100 segments	200	Yes
Helsinki University Hospital EEG	79	19	International 10–20 system	Scalp EEG	460 events	256	No
Kaggle American Epilepsy Society Seizure Prediction Challenge	2 patients 5 dogs	16	Intracranial	Intracranial EEG	111 events	400	No
Siena Epilepsy Dataset	14	20–29	International 10–20 system/nomenclature	Scalp EEG	47 events	512	No

### Pre-processing

**Fig. 7:**
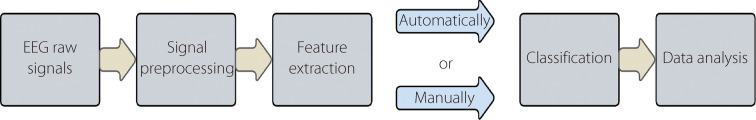
EEG signal processing paradigm

Processing of EEG signals is critical for predicting and detecting epileptic seizures. Typically, EEG signals exhibit amplitudes ranging from 10 µV to 100 µV and frequencies extending from 1 to 100 Hz. Spectral characteristics derived from raw signals using Fourier or WTs can assist in diagnosing neural disorders and decoding brain activity. Spectral analyses rely on predefined frequency bands. A typical approach for processing EEG signals involves the steps shown in Fig. 7.

Initially, brain waves are recorded, and raw EEG signals are preprocessed to remove undesirable artifacts and noise. Because EEG signals only represent approximately 5% of the native brain signals, preprocessing crucially enhances signal fidelity by employing high-pass, low-pass, and notch filters to eliminate noise and artifacts.

A bandpass filter permits signals within a defined frequency range to pass through while attenuating the rest. This is essential for isolating the relevant frequency bands. High-pass filtering permits frequencies above a cutoff point to pass while attenuating lower frequencies, effectively removing low-frequency components from the signal. Low-pass filters allow frequencies below a cutoff point to pass through while attenuating higher frequencies, making them useful for smoothing or removing high-frequency noise. Notch filters, also known as band-stop or band-reject filters, target a narrow range of frequencies for attenuation while allowing all other frequencies to pass through, making them ideal for eliminating interference at specific frequencies. Filtering is efficient when the frequency ranges of the signals are distinct without overlapping; however, alternative artifact-removal methods are necessary when spectral overlap occurs. These methods include WT, regression, and EMD. Additionally, mixed approaches such as BSS-SVM, wavelet-BSS, and EMD-BSS are employed for enhanced artifact removal. Fluctuating voltage amplitudes in raw EEG signals hinder the effectiveness of computer-aided detection systems for identifying and predicting epileptic seizures. Standardization techniques, including Z-score normalization, are recommended to mitigate this. Additionally, EEG signal storage and processing require significant memory. Downsampling EEG signals reduces the sampling frequency, thereby decreasing the storage space required. Windowing or segmenting EEG signals constitutes the final processing step. Segmentation breaks down EEG signals into smaller and more detailed segments, thereby facilitating the extraction of more meaningful features from each part of the signal.

## Feature extraction and selection

**Fig. 8:**
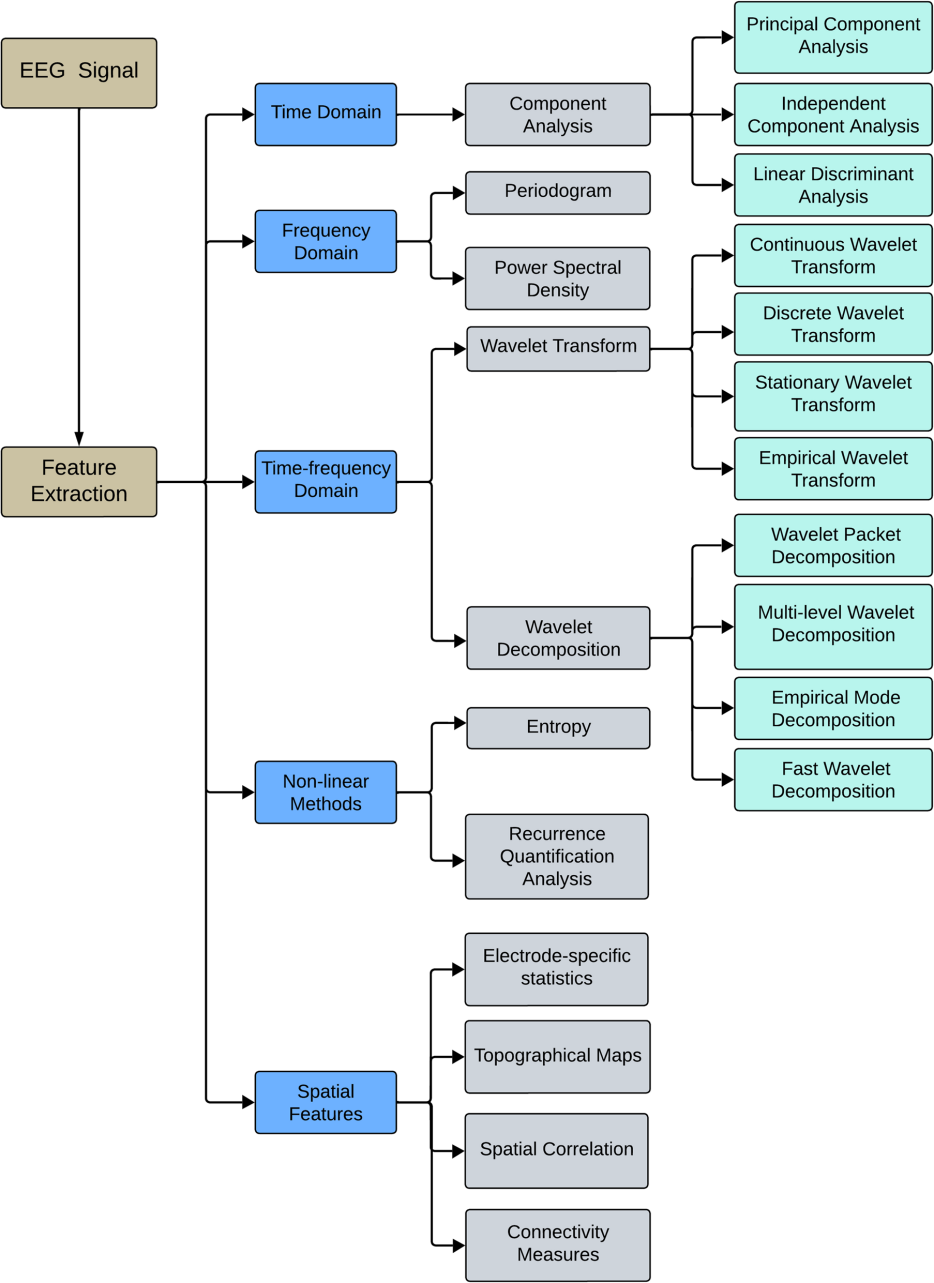
Feature extraction methods

Once pre-processing has produced signals free from noise and artifacts, the next step involves extracting effective and meaningful characteristics from the EEG signals. Feature extraction entails deriving pertinent information from raw EEG signals to represent them in a form suitable for ML algorithms. These methods aim to capture the distinctive patterns and characteristics of EEG signals indicative of seizure activity. Before feature extraction, the EEG signals are divided into segments with predefined durations. For each segment, feature extraction techniques can be applied to compute features synchronized with specific events [51]. Commonly used features include time-domain metrics, such as variance, skewness, and mean; frequency-domain characteristics, such as power spectral density (PSD); and time-frequency characteristics, such as the short-time Fourier transform (STFT) and WT coefficients [52]. The various techniques used for feature extraction from brainwave signals are presented in Fig. 8. The most prevalent methods include principal component analysis (PCA), independent component analysis (ICA), wavelet packet decomposition (WPD), autoregressive (AR), WT, and fast Fourier transform (FFT), of which Table 2 presents a comparative analysis [53, 54]. Subsequently, feature selection techniques determine the most informative features while reducing the dimensionality and computational complexity. This guarantees that the model focuses on the most discriminative information in the data, thereby improving its ability to distinguish between the seizure and non-seizure states.

**Table 2:** Comparing various feature extraction techniques

Method	Strength	Limitation
ICA	Reduces the dimensionality of data by transforming it into a set of statistically independent components Exhibits high performance with large datasets. Robustness to Gaussian noise	Works better with non-Gaussian sources, and its performance may degrade if the source signals have Gaussian distributions Computationally intensive The order of the extracted components is arbitrary and can change with each run, making it challenging to interpret and compare results consistently
WT	Capable of analyzing signals with discontinuities using various window sizes. Provides a multi-resolution analysis of the signal, enabling the examination of both low-frequency and high-frequency components simultaneously Various types of wavelets	Choosing the appropriate wavelet basis function can be challenging and may require domain knowledge Suffers from boundary effects, where the edges of the signal may produce artifacts, potentially affecting the analysis of features near the boundaries Uncertainty
AR	Excels at capturing the temporal dynamics of EEG signals It analyzes how past activity influences the present, revealing the short-term dependencies within the data Suitable for real-time applications where quick processing is essential	Assumes that the underlying process generating the EEG signal is stationary Focus on linear relationships between past and present values
PCA	While not directly interpretable in the original feature space, principal components can sometimes be linked back to meaningful aspects of the data through domain knowledge. Tends to prioritize features with a stronger signal-to-noise ratio	Assumes a linear relationship between the original features Sensitive to the scaling of the original features
FFT	Can be used to isolate specific frequency bands of interest and filter out noise at other frequencies	Assumes the signal being analyzed is stationary, meaning its statistical properties don’t change over time The choice of window function used during FFT analysis can impact the resulting frequency spectrum Transforms a signal from the time domain to the frequency domain. While it provides information about the constituent frequencies, it loses the exact timing information of these frequencies within the original signal
WPD	Can analyze the non-stationary signals. Decomposes a signal into a hierarchy of subbands in both time and frequency domains	Increases computation time

### Time domain

Time domain techniques directly analyze EEG data in the time domain by focusing on their amplitude and temporal characteristics. These methods provide a straightforward and intuitive approach to understanding fundamental brain activity patterns by extracting relevant information from a raw signal based on its time domain characteristics.

Common time domain characteristics include statistical measures, such as variance, mean, skewness, and kurtosis, which offer insights into the signal’s central tendency, variability, and shape. Other features may encompass temporal parameters, such as rise time, fall time, and duration, which capture aspects of signal dynamics over time. Time domain feature extraction is critical for acquiring the essential characteristics of a signal pertinent to a given task, such as the identification of specific events, patterns, or abnormalities.

### Frequency domain

Frequency domain techniques focus on analyzing the frequency content of EEG waveforms rather than their time-based characteristics. Methods, such as Fourier transform, WT, and spectrogram analysis, transform raw EEG data from the temporal domain to the spectral domain. This transformation decomposes the signal into its individual frequencies, thereby exposing the amplitude and phase of each frequency component. Frequency domain analysis is vital for identifying periodicities, filtering noise, and understanding the underlying structures of complex signals. Spectral methods are commonly classified into parametric and nonparametric categories. Nonparametric techniques directly estimate the spectral characteristics of a signal from the data. The periodogram is a common nonparametric method that estimates the PSD of a signal using a Fourier transform. The Welch method is another popular approach that enhances the periodogram by averaging the PSD estimates of the overlapping signal segments to reduce variance. These techniques are widely used because of their simplicity and ability to provide insights into the frequency content of signals without requiring prior knowledge of the signal structure. Despite these advantages, these techniques are sometimes imprecise, particularly when estimating the spectral density of signals with complex structures. This can be addressed using parametric methods. Parametric approaches assume that a signal can be modeled using a finite number of parameters. These techniques rely on the premise that a signal is generated by a linear system driven by white noise. Using parametric methods, such as AR, moving average (MA), or autoregressive moving average (ARMA), can more accurately estimate the PSD for nonparametric methods, particularly for short data records.

The model parameters are identified using techniques such as the Yule-Walker equation or Burg method. Parametric approaches are advantageous because they provide smoother and more precise spectral estimates, which are particularly useful when dealing with complex or low signal-to-noise ratio environments. In an AR model, each value in a time series is represented as a linear combination of past values weighted by coefficients known as AR parameters. Mathematically, an AR model of order* p* is expressed as a linear regression of the current value on the previous *p* values, which can be defined as [55]:

1 $${x}_{t}=c +{\sum }_{k=1}^{p}{\rho }_{k} {x}_{t-k}+ {\epsilon }_{t}$$

where *c* is a constant, ϵ_*t*_ is white noise, and *ρ*_1_,..., ρ_*a*_ are the model parameters.

MA models are a class of parametric models widely employed in time-series analysis and signal processing. The MA model represents each value in a time series as a linear combination of past white noise terms, where the order of the model determines the number of past white noise terms included in the combination.

Mathematically, an MA model of order *q* is expressed as a linear regression of the current value on the past *q* white-noise terms. They are particularly useful for smoothing short-term fluctuations or noise in a time series, making them effective for filtering and denoising signals. ARMA methods integrate AR and MA elements to capture both temporal dependencies and random fluctuations in a time series. An ARMA model presents the current value in a time series as a linear combination of its past values (AR component) and the past values of a stochastic error term (MA component). Mathematically, an ARMA model of order (*p*, *q*) is defined by *p* AR terms and *q* MA terms, as follows:

2 $${x}_{t}=c+{\sum }_{k=1}^{p}{\uprho }_{k}{x}_{t-k}+{\upepsilon }_{t}+{\sum }_{k=1}^{q}{\uptheta }_{k}{\upepsilon }_{t-k}$$

*ρ*_1_,..., ρ_*p*_ are AR coefficients, and *θ*_1_​,..., *θ*_q_ are MA coefficients.

Estimating the parameters of an ARMA model typically involves techniques such as maximum likelihood estimation or least-squares estimation.

### Time-frequency domain

Time-frequency domain techniques are a class of signal processing methods that simultaneously analyze the time and frequency characteristics of signals and provide insights into their dynamic behavior over time. Unlike traditional time- or frequency-domain methods, which focus solely on one aspect of the signal, time-frequency methods offer a more comprehensive view by capturing how the signal’s frequency components evolve over time. Common time-frequency analysis techniques include the spectrogram, STFT, and continuous wavelet transform (CWT). STFT divides signals into brief segments and computes the Fourier transform for each segment, providing a time-varying representation of the signal’s frequency content. The CWT offers variable time and frequency resolutions, rendering it appropriate for the analysis of non-stationary signals with time-varying frequencies. The spectrograms provide a graphical representation of the energy distribution of the signal across various frequencies and times, allowing the identification of transient events and frequency modulations.

Time-frequency domain methods are widely used in many applications, including biomedical signal processing, audio analysis, and communication systems, where capturing both time and frequency characteristics is essential for understanding the signal behavior and extracting meaningful features.

### Spatial features

Spatial features in EEG signal processing refer to the characteristics extracted from the spatial distribution of electrical activity measured by electrodes placed on the scalp. These characteristics offer information about the spatial organization and patterns of brain activity and are crucial for understanding brain function and dysfunction. Common spatial features include the following:

- Topographical maps: Visualization of the voltage distribution across the scalp, often represented as color-coded maps.
- Electrode-specific statistics: Statistical measures (e.g., mean and variance) are calculated for individual electrodes.
- Spatial correlation: Measures the degree of similarity or correlation between electrode pairs, indicating functional connectivity.
- Topographical power distribution: The distribution of power across different frequency bands across scalp regions.
- Connectivity measures: The functional connectivity between brain regions is often assessed using measures such as coherence, phase synchronization, or imaginary coherence.

These spatial features are essential for tasks such as localizing brain activity, identifying brain networks, and understanding the spatial dynamics of cognitive processes or neurological disorders.

### Nonlinear methods

#### Recurrence quantification analysis

Recurrence quantification analysis (RQA) is a powerful method used to analyze complex dynamic systems, particularly nonlinear time-series analysis and chaos theory. RQA provides a comprehensive set of measures for characterizing the properties of recurrences within a phase space reconstructed from time-series data. These measures include the recurrence rate, determinism, and entropy, which capture different aspects of the system dynamics, such as its regularity, predictability, and complexity. RQA is applied in diverse fields, including biology, physics, finance, and engineering, being used to analyze and understand the behavior of complex systems that exhibit nonlinear and chaotic dynamics. By quantifying the recurrence properties of a system, RQA enables researchers to uncover hidden patterns, transitions, and critical behaviors, thereby offering valuable insights into the underlying mechanisms governing the system’s evolution.

####  Entropy

Entropy, a primary concept in information theory and statistical mechanics, measures the uncertainty or randomness of a system [56]. In the context of information theory, entropy provides a quantitative assessment of the information present in a random variable, representing the average amount of surprise or unpredictability associated with observing outcomes. The higher the entropy, the more uncertain the outcomes and vice versa. In statistical mechanics, entropy characterizes the disorder or randomness of a physical system and reflects the number of microstates corresponding to a given macrostate. Generally, approximate entropy is used to measure the uncertainty and consistency of EEG signals as well as the extent of instability variations within the signal [57–59]. The approximate entropy expresses the logarithmic probability that a signal with *N* sample points repeats itself within a tolerance of *r* for* m* points and subsequently *m* + 1 points. For a specific time series *y*(*i*) of length $L$, $L-m+1$ vectors $Y\left(1\right)$, $Y\left(2\right)$..., $Y(L-m+1)$, were constructed.

Approximate entropy is defined as follows [58]:

3 $$ApEn\mathit{\left({m,\;r,\;N}\right)}\mathit=\phi m\mathit{\left(r\right)}\mathit-\phi m\mathit+\mathit1\mathit{\left(r\right)}$$

where *ϕm(r)* is the average logarithmic probability that sequences of length *m* remain similar within tolerance *r*, $m$ is the embedding dimension, *r* is the tolerance threshold, and $N$ is the length of the time series.

4 $${\upphi }^{m}\left(r\right)=\frac{1}{N-m+1}{\sum }_{i=1}^{N-m+1}\text{ln}{C}_{i}^{m}\left(r\right)$$

C_*m*_ is a correlation integral indicating the probability of vector $Y(i)$, which remains similar to $Y(j)$ within tolerance limit *r*.

5 $${C}_{i}^{m}\left(r\right)=\frac{\text{Number of }j\text{ where }d\left({Y}_{i},{Y}_{j}\right)\le r}{N-m+1}$$

$d\left({Y}_{i},{Y}_{j}\right)$ is the distance between vectors (e.g., Chebyshev, Euclidean).

### Feature selection

**Fig. 9:**
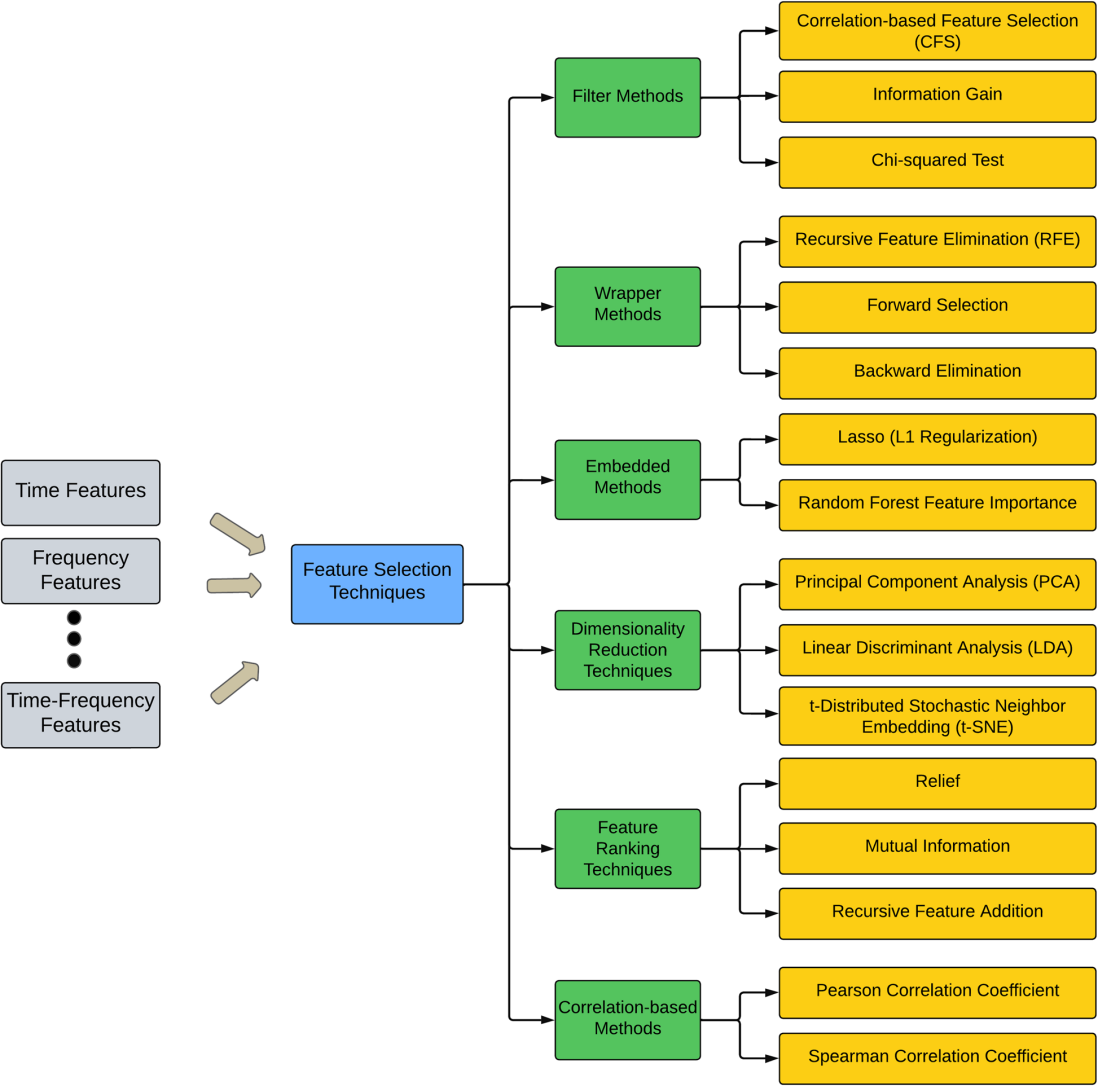
Feature selection techniques

Feature selection is essential in ML and data analysis, particularly in scenarios in which the dataset contains an extensive set of characteristics. This involves selecting a group of pertinent features from the initial set to enhance model performance, mitigate overfitting, and improve interpretability. Feature selection aims to determine the optimal, most informative, and discriminative features that contribute most to the model’s predictive power while discarding redundant or irrelevant features. By selecting only the most relevant characteristics, feature selection can lead to simpler and more efficient models, reducing computational complexity and improving the generalization of new data. Feature selection can be classified into six types: the wrapper, filter, embedded, dimensionality reduction, feature ranking, and correlation-based methods (Fig. 9). Filter methods evaluate the relevance of features separately from the learning model and select features based on statistical or heuristic criteria.

Wrapper methods assess feature groups by training a model and measuring its performance using various feature combinations. Embedded methods integrate feature selection into the model training procedure, typically by including feature selection when optimizing the learning algorithm. Dimensionality reduction methods, such as t-distributed stochastic neighbor embedding, linear discriminant analysis (LDA), and PCA, can be used to reduce the dimensionality of EEG signals while retaining relevant information. These methods project the original EEG signals onto a lower-dimensional space, making it easier for classification algorithms to learn discriminative patterns. Feature ranking techniques, such as relief, mutual information, and recursive feature addition, rank features based on their relevance to the target class. These techniques assign a score to each feature, indicating its importance for classification and allowing researchers to select the top-ranked features for model training.

## Epileptic seizure detection and prediction approaches

Classifying epileptic seizures is a fundamental step in the diagnostic process. By utilizing sophisticated algorithms and ML methods, classification models can analyze intricate patterns within brainwave signals to differentiate between seizure and non-seizure activities. These models are trained on diverse datasets containing EEG recordings from individuals with epilepsy to capture the complex dynamics of seizure events. The classification process involves identifying characteristic features, such as the spike-and-wave patterns, spectral changes, and temporal dynamics unique to seizure activity. Among the various available classification approaches, the most frequently utilized algorithms are ANNs, k-nearest neighbors (k-NNs), SVMs, RF, hidden Markov models, and DL methods. EEG classification algorithms can be categorized as matrix and tensor classifiers, adaptive classifiers, DL, and transfer learning methods. Adaptive classifiers adjust their parameters incrementally over time as new data become available, making them effective for tracking variations in the distribution of features, particularly in dynamic signals, such as EEG. Transfer learning utilizes knowledge acquired by addressing one problem to solve another that is closely related. Instead of training a model from scratch, transfer learning involves leveraging models, typically pretrained on large datasets, and fine-tuning them on a smaller dataset specific to the target task. Matrix and tensor categories operate on multidimensional data representations, such as matrices and tensors. These classifiers are designed to handle complex data structures with multiple dimensions, making them suitable for tasks where the input data exhibit intricate relationships and interactions. Matrix classifiers typically operate with two-dimensional matrices, whereas tensor classifiers extend this concept to higher-dimensional tensors. ML- and DL-based models have proven to be effective for epileptic seizure detection and prediction, offering promising avenues for advancing diagnosis and patient care.

**Fig. 10:**
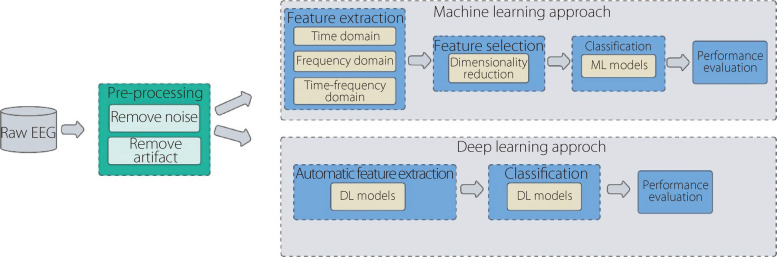
Comparison of ML and DL techniques for identifying epileptic seizures

A comparison of the ML and DL methods for epileptic seizure identification is shown in Fig. 10. These models leverage advanced algorithms to analyze EEG signals, which are highly informative for detecting epileptic activity.

Traditional ML approaches such as k-NN, SVMs, and RF, which extract pertinent characteristics from EEG signals and train classifiers to distinguish between seizure and non-seizure segments, have been widely applied for seizure identification. In contrast, DL techniques, particularly CNNs and RNNs, have shown remarkable success in learning hierarchical and temporal representations directly from raw EEG signals. DL models can automatically extract intricate patterns and temporal dependencies, enabling more accurate and robust seizure detection and prediction. Moreover, the emergence of DL has enabled the advancement of end-to-end systems that can seamlessly integrate feature extraction and classification, thereby improving their performance and efficiency.

### Conventional ML techniques for seizure identification

**Fig. 11:**
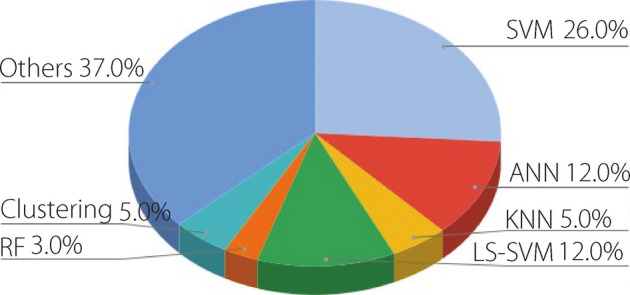
Percentages of conventional ML techniques used in epilepsy studies

AI algorithms are widely employed as classifiers in automated epilepsy identification systems [60]. The examination of EEG signals is crucial for detecting and predicting epileptic seizures. Various artifact removal techniques are utilized to eliminate signal noise and artifacts. Additionally, conventional methods for feature extraction are applied to extract features from the data, statistically analyze, rank, and select them as inputs for ML classifiers. Several classification techniques have been proposed, including LR, ANNs, RFs, fuzzy logic, k-NNs, and SVMs with different kernel functions. Figure 11 shows the proportion of traditional methods employed by researchers. Table 3 presents an overview of the literature that employs ML algorithms with diverse feature extraction methods. Table 4 presents a comparison of the predominant machine-learning algorithms employed in epileptic seizure identification. A comparison to consider the strengths and weaknesses of each was conducted using the most prevalent and widely used classifiers.

**Table 3:** Studies using ML algorithms

Reference	Year	Used dataset	Pre-processing, feature extraction	Classifier	Result
[61]	2025	UCI	Segmentation SMOTE balancing PCA, DWT	SVM DT RF k-NN	ACC = 97.30%, F1-score = 93.08%, AUC = 99.62% ACC = 94.48%, F1-score = 85.87%, AUC = 83.24% ACC = 97.17%, F1-score = 92.72%, AUC = 99.56% ACC = 92.13%, F1-score = 76.03%, AUC = 88.48%
[62]	2025	Bonn	Low-pass filter, Noise injection, PSD features	RF k-NN MLP	ACC = 100%, SEN = 100% ACC = 100%, SEN = 99.75% ACC = 100%, SEN = 99.67%
[63]	2025	Animals’ EEG dataset	PCA, Hjorth’s parameters, Daubechies DWT coefficients	SVM	SEN = 90.00%, SPE = 74.00%
[64]	2025	Clinical	Statistical features	DT LR NB SVM Tree Bagger k-NN	ACC = 77.70%, SEN = 86.00%, SPE = 60.00% ACC = 59.20%, SEN = 60.00%, SPE = 58.00% ACC = 80.00%, SEN = 89.00%, SPE = 63.00% ACC = 80.80%, SEN = 91.00%, SPE = 60.00% ACC = 91.50%, SEN = 97.00%, SPE = 81.00% ACC = 69.20%, SEN = 84.00%, SPE = 40.00%
[65]	2024	EPILEPSIAE	CNN-based artifact removal, 5 s sliding window, 59 univariate linear features × 19 channels	LR 15 SVMs ensemble 15 SNNs ensemble	SS = 0.13 + 0.26, FPR/h = 0.36 + 0.40 SS = 0.13 + 0.21, FPR/h = 0.73 + 0.85 SS = 0.12 + 0.20, FPR/h = 0.48 + 0.66
[66]	2024	Hauz Khas, Bern Barcelona, Bonn	Filtering, Huguchi, Katz, spectral entropy, PSD	RF AB GB ET ARF SVM NB	ACC = 91.00%, SEN = 94.20%, SPE = 88.20% ACC = 91.20%, SEN = 92.10%, SPE = 90.00% ACC = 91.20%, SEN = 94.20%, SPE = 88.30% ACC = 94.30%, SEN = 94.30%, SPE = 94.30% ACC = 89.00%, SEN = 96.50%, SPE = 84.00% ACC = 78.30%, SEN = 76.40%, SPE = 60.30% ACC = 74.30%, SEN = 76.40%, SPE = 72.40%
[67]	2024	EPILEPSIAE	CNN-based artifact removal. Time domain and frequency domain features, Z-score	SVM	SS = 0.75 + 0.33 FPR/h = 1.03 + 1.00
[68]	2024	Helsinki CHB-MIT	Savitzky-Golay filter, WSD, DWT, 6 statistical features	SVM	ACC = 92.82%, SEN = 94.65%, SPE = 90.80% ACC = 90.26%, SEN = 97.50%, SPE = 85.83%
[69]	2023	Bern Barcelona	EMD-DWT-based decomposition, log-energy entropy	SVM k-NN	ACC = 58.33%, SEN = 60.87%, SPE = 56.76% ACC = 75.00%, SEN = 77.78%, SPE = 72.73%
[70]	2023	CHB-MIT	Filtering, segmentation, parametric PSD	SVM k-NN LDA	ACC = 99.95%, SEN = 99.90%, SPE = 100% ACC = 99.80%, SEN = 99.80%, SPE = 99.80% ACC = 99.05%, SEN = 99.00%, SPE = 99.00%
[71]	2023	Bonn	Elliptical mode decomposition DWT, log-energy entropy	k-NN SVMA	ACC = 75.00%, SEN = 77.78%, SPEC = 72.73% ACC = 58.33%, SEN = 60.87%, SPEC = 56.76%
[72]	2023	CHB-MIT	Channel selection, FIR-based bandpass filter, 2nd order Butterworth filter, notch filter, ICA for rejecting large artifact timepoints, FFT, WT, mean, standard deviation	SVM	ACC = 91.00%, SEN = 98.00%, SPEC = 84.00% F1-score = 91.00%
[73]	2022	CHB-MIT	Filtering, Tunable Q-WT	SVM RF	ACC = 90.40%, SEN = 89.20% ACC = 93.00%, SEN = 91.50%
[74]	2021	CHB-MIT	ICA, 8 statistical features, PSD, pattern adaptive WT	Fuzzy classifier	ACC = 96.48%, SEN = 96.52%, SPE = 95.34%
[75]	2021	Bonn	FDT, DWT, FFT + fuzzification	Fuzzy DT Fuzzy RF	ACC = 99.50%, SEN = 99.60%, SPE = 99.30% ACC = 99.30%, SEN = 99.40%, SPE = 99.50%
[76]	2019	Bonn	DWT	Particle swarm optimization + SVM	ACC = 99.38%
[77]	2019	Bonn	Segmentation, FFT, DWT, statistical features, InfoGain	Least square SVM	ACC = 100%
[78]	2019	CHB-MIT	PCA, poincare section, statistical features	SVM + Naïve Bayes	ACC = 96.28%, SEN = 97.50%, SPEC = 94.50%
[79]	2019	Bonn	DWT, 8 types of entropies, ANOVA	LS-SVM	ACC = 99.50%, SEN = 100%, SPE = 99.40%
[80]	2019	Bonn Freiburg	Segmentation, DWT, 5 statistical features	RF	ACC = 95.00%, SEN = 99.74%

**Table 4:** A comparison between the most used classification techniques

Classifier	Strength	Weakness
SVM	Provides good generalization. Operates better than other linear classifiers	High computational complexity
k-NN	Easy to comprehend and implement Easy to debug	Large training sets may result in inadequate runtime performance. Memory intensive Curse of dimensionality Tends to favor majority classes in imbalanced datasets, leading to biased predictions
ANN	Robustness to noise Parallel processing Adaptability to various data types. Non-linearity	Complex to design and tune Sensitive to hyperparameters Require large amounts of training data
RF	Reduces overfitting and is efficient with large datasets Robust	Not suitable for imbalanced data Lack of interpretability
LDA	Low computational requirements Simple to use Provides good results	Limited for non-normal data Not robust with small sample sizes. Assumes normality and equal covariance
Naïve Bayes	Robust to irrelevant features Works well with categorical and continuous data	Assumes independence among features. Sensitive to feature correlation

### DL approaches for seizure identification

**Fig. 12:**
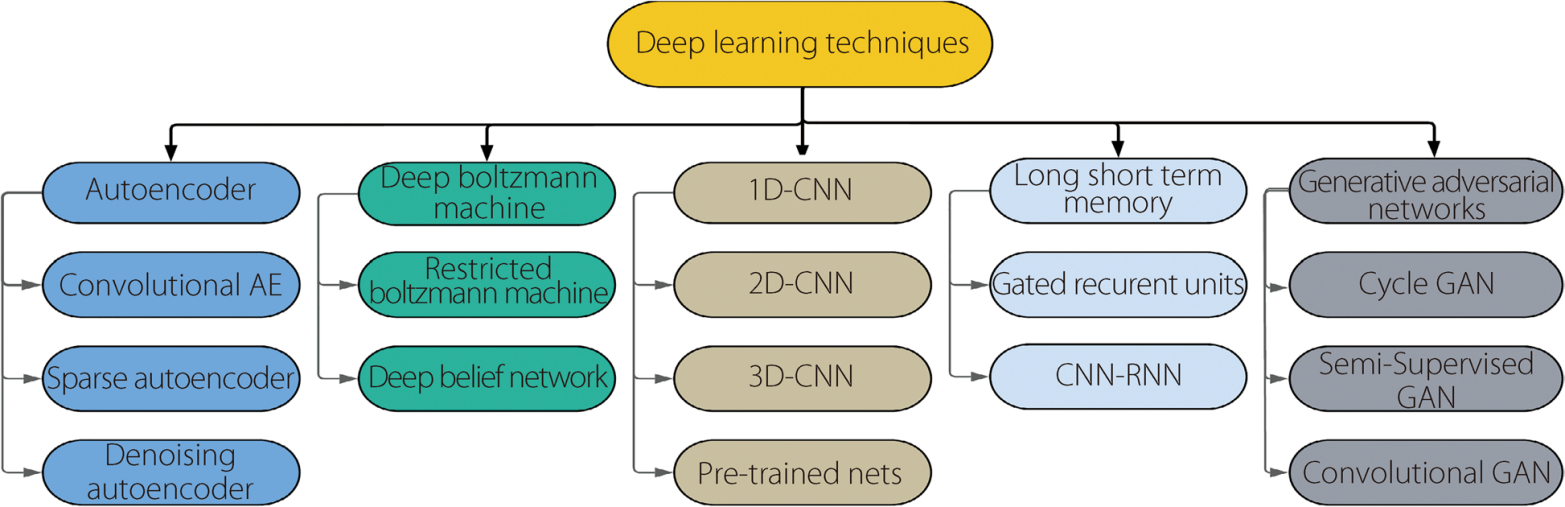
DL methods used for epileptic seizure detection

DL has emerged as a transformative technology in the medical domain, offering innovative solutions across various healthcare applications, including disease detection, diagnosis, and prognosis. In epilepsy detection and prediction, DL techniques have shown remarkable potential in aiding clinicians with more accurate and timely assessments. By leveraging large volumes of EEG data, DL algorithms can automatically learn hierarchical representations and features indicative of epileptic activity, enabling them to identify seizures with elevated sensitivity and specificity. Moreover, DL algorithms can analyze long-term EEG recordings to predict seizure onset, providing patients with valuable insights into their condition and potentially allowing for early intervention strategies. The DL models’ capacity to process and interpret vast amounts of medical data could potentially revolutionize epilepsy management, introducing more personalized and effective treatment approaches. Many researchers have focused on using brainwave signals for epilepsy detection and prediction using DL methods, whereas, other than EEG, few have employed modalities such as sMRI, fMRI, and PET. Table 5 provides a summary of the relevant studies utilizing various PET and MRI neuroimaging modalities. The DL architectures shown in Fig. 12 facilitate the recognition of epileptic seizures. Notably, among these architectures, 2D-CNN and 1D-CNN are extensively utilized for this purpose.

**Table 5:** Summary of related work utilizing medical imaging techniques and DL

Reference	Dataset	Modality	Processing	Input network	DNN	Classifier
[81]	Clinical	MRI	Normalization, skull-stripping, bias field correction, intensity, patch extraction	Patches	CNNs	Softmax
[82]	Clinical	Diffusion MRI	HARDI and NODDI to generate the connectivity matrix	Connectivity matrix	Inception ResNetV2	Softmax
[83]	HCP dataset	MRI	Create LF images from HF references	Created LF and HF images	Aniso-U-Net	-
[84]	Clinical	DWI	Series of different preprocessing procedures	14 ESM	DCNN	Softmax
[85]	ECP project	sMRI, rsfMRI, task-fMRI, PDC data	Features from sMRI, ROI correlation matrices derived from rs-fMRI, task fMRI, and PDC preprocessed MRI	Features from sMRI, ROI correlation matrices derived from rs-fMRI and task fMRI, and PDC	Multi-channel DNN	Softmax
[86]	Inselspital	MRI	Generation of ground truth through skull stripping, resampling, FreeSurfer, cropping, and rescaling	Preprocessed MRI	3D-CNN	Softmax
[87]	SCTI-MST	MRI	Noise reduction with BM3D algorithm, skull-stripping, FCD lesion segmentation	256 × 256 image size	FCN	Sigmoid
[88]	Clinical	DWI, fMRI	Different methods	DWI streamline coordinate	DCNN-CL-ATT	Softmax
[89]	Clinical	PET	ROI, normalization, AAL, CNNI, downsampling, NNI (3D images)	2D ROI, 3D ROI	2D-ResNet50, 2D-VGG16, 2D-InceptionV3, 3D-SVGG-C3D	Sigmoid
[90]	Clinical	PET	Ordered subset expectation maximization, DA radionics features	Non attenuation corrected 2D PET image slices	Deep-DAC	Tanh
[91]	Clinical	MRI	MRI	3D patches	Two-stage CNNx cascade	Softmax
[92]	Clinical	EEG-fMRI	Filtering, ICA, BCG, GLM, MCS	IEDs	ResNet	Triplet Softmax
[93]	Clinical	sMRI, rs-fMRI	Resizing, denoising	3D scans	VoxCNN-B	Softmax
[94]	Clinical	MRI	Scaling down	Raw MRI data	3D-CNN	Softmax
[95]	Clinical	MRI	Creation of binary masks for dimensionality reduction of the connectivity matrix	SZ and SZF masks	2D-CNN	Softmax

#### CNNs

**Fig. 13:**
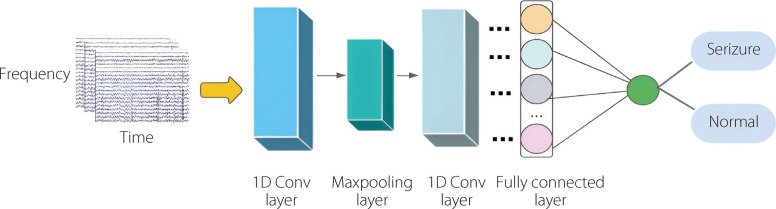
Standard workflow employing 1D CNNs for seizure detection

CNNs [96] are a type of DL algorithm that has revolutionized the field of computer vision. It extends the capabilities of a multilayer perceptron (MLP) by extracting characteristics from data using convolutional operations. Unlike MLPs, CNN neurons are connected only to a localized region, rather than being fully connected. This localized connectivity mirrors the hierarchical processing of information in the brain, enabling CNNs to effectively capture local features. CNNs are composed of several layers, including convolutional, pooling, and fully connected layers. Convolutional layers use filters to process input data, extract features, and capture local patterns, such as shapes, edges, and textures. Pooling layers diminish the dimensionality of the data, enhance network efficiency, and improve resilience to input variations. The fully connected layers integrate the extracted features to make final predictions. Additionally, CNNs share weights among specific neurons within the same layer, aiding in learning and capturing particular features while reducing the number of parameters and enhancing generalization [97]. The hierarchical architecture of CNNs enables them to acquire progressively abstract and intricate representations of input data through each successive layer. This powerful capability to learn the spatial hierarchies of features automatically and adaptively has led CNNs to be the backbone of numerous applications ranging from medical image analysis to autonomous driving and beyond. CNNs have emerged as a mainstream method for EEG-based epileptic-seizure identification, demonstrating excellent performance in feature extraction and classification. Figure 13 shows the standard workflow utilizing 1D-CNNs. Das et al. [98] utilized EMD to decompose brainwave signals into six intrinsic mode functions (IMFs).

**Fig. 14:**
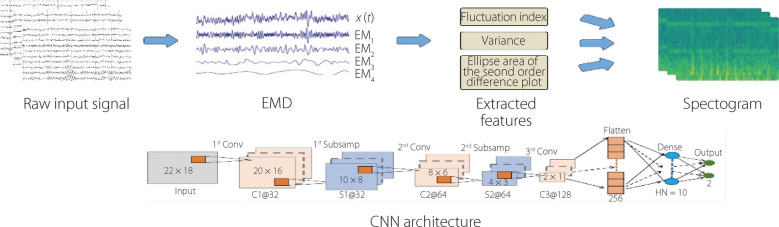
CNN-based analysis of 2D-EEG spectrograms for ES detection

Three distinct features are derived from each IMF: the ellipse area of the second order, variance, and fluctuation index. The extracted characteristics from all channels are then organized into two composite formats: unidimensional and image-like. To detect epileptic seizures, the effectiveness of CNNs was evaluated using an extracted spectrogram and employing a 1D version of the CNN for the unidimensional feature form. The experiment encompassed datasets from both the CHB-MIT and Prince Hospital Khulna (PHK), Bangladesh, comprising EEG signals from patients with epileptic seizures. The CNN model utilizing 2D features as shown in Fig. 14, outperformed the other models relying on 1D features, attaining accuracies of 99.78% for the CHB-MIT and 95.26% for the PHK datasets.

**Fig. 15:**
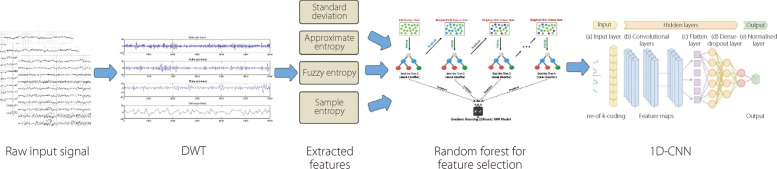
CNN-based 1D-EEG analysis in ES detection

**Fig. 16:**
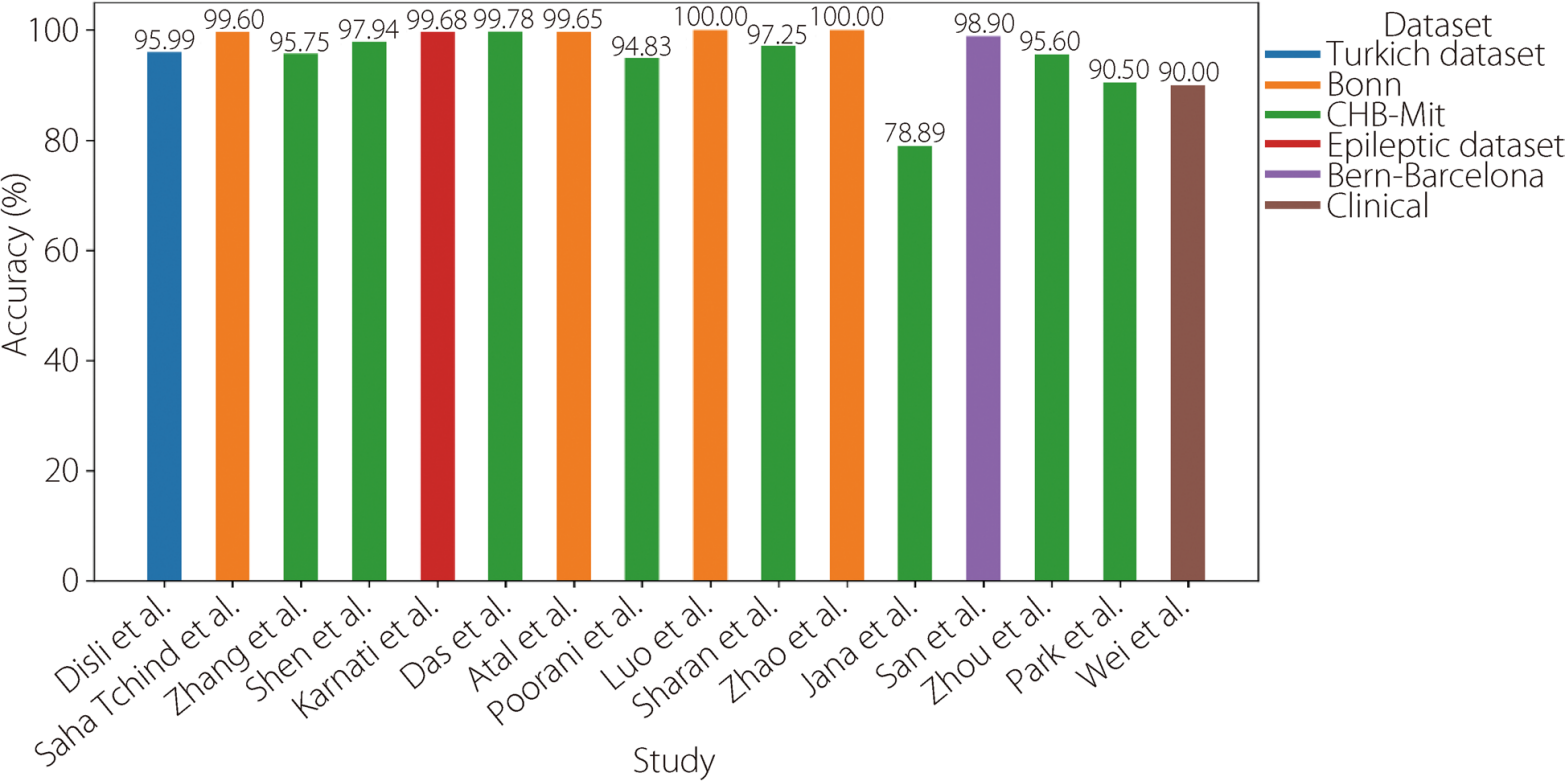
Analysis of the accuracy of results from various studies employing CNN models for epilepsy identification

Toraman [99] adopted an approach in which preictal and interictal events were segmented into five-second intervals. These segmented signals were then transformed into spectrograms, and the resulting spectrograms were classified using three distinct pretrained CNN models (VGG19, ResNet, and DenseNet). The highest accuracy score of 91.05% is achieved for the CHB-MIT dataset. Chen et al. [100] adopted a method based on feature fusion and selection. The proposed architecture is illustrated in Fig. 15. Initially, they extracted mixed features, including standard deviation, fuzzy entropy, sample entropy, and approximate Entropy, from the sub-bands generated through DWT decomposition. Subsequently, they employed an RF algorithm to select pertinent features, followed by the use of a CNN to classify the epileptic signals. Their approach was empirically assessed using the Bonn and New Delhi datasets. The proposed model demonstrated exceptional performance, achieving an accuracy of 99.9%, specificity of 99.8%, precision of 99.81%, and sensitivity of 100% for the Bonn dataset. Similarly, in the interictal classification scenario of the New Delhi dataset, the model achieved perfect scores with 100% accuracy, 100% precision, 100% specificity, and 100% sensitivity [100]. Table 6 and Fig. 16 provide an overview of the literature on CNNs for seizure detection and prediction.

**Table 6:** Available literature using CNNs

Reference	Year	Dataset	Pre-processing	Technique	Accuracy	Sensitivity	Specificity
[101]	2025	Turkish Epilepsy Dataset	Normalization	CWT + 2D-DepthwiseConv network	95.99%	94.28%	97.29%
[102]	2025	Bonn NSC-ND	Normalization	Log-Mel spectrogram + 2D CNN	99.60% 93.33%	99.33% 90.00%	99.67% 95.00%
[103]	2025	CHB-MIT	Normalization Segmentation	Inception CNN	95.75%	94.60%	-
[104]	2024	CHB-MIT	6th-order Butterworth filter	STFT + 29-layer Google-Net	97.94%	98.90%	-
[105]	2024	Bonn NSC-ND Epileptic dataset	Windowing Normalization	CWT + dilated CNN	97.61% 96.11% 99.68%	98.00% 95.00% 100.00%	- - -
[98]	2024	PHK CHB-MIT	Segmentation Filtering Normalization EMD	3 statistical features + 2D-CNN	95.26% 99.78%	- -	- -
[106]	2024	Bonn	MBBF	GPSO optimized + 1D-CNN	99.65%	-	-
[107]	2023	CHB-MIT	Normalization	1D-CNN	94.83%	90.18%	99.48%
[108]	2022	Bonn	EMD WOG	2D-CNN	100.00%	-	-
[109]	2020	CHB-MIT	Segmentation WT	1D-CNN	97.25%	-	-
[110]	2020	Bonn	Segmentation normalization	1D-CNN	100.00%	100.00%	100.00%
[111]	2020	CHB-MIT	Butterworth filter	Spectrogram + 1D-CNN	78.89%	66.67%	91.11%
[112]	2019	Bern-Barcelona	FT, WT, EMD	1D-CNN	98.90%	-	-
[113]	2018	CHB-MIT Freiburg	FFT	1D-CNN	95.60% 96.70%	- -	- -
[114]	2018	SNUH- HYU CHB-MIT	Low-pass filter	1D-CNN + 2D-CNN	90.50% 92.76%	- -	- -
[115]	2018	Clinical	Segmentation 3D image reconstruction	3D-CNN	90.00%	88.90%	93.78%

#### RNNs

RNNs [116] represent a pivotal innovation in AI and ML. Unlike traditional feed-forward NNs, RNNs have demonstrated dynamic temporal behavior, rendering them exceptionally well-suited for sequential data tasks. RNNs contain internal memory that enables information regarding previous inputs to be retained. This memory mechanism enables them to identify dependencies and patterns in sequential data, including time series, natural language, and audio signals. RNN architectures comprise recurrent connections that form loops, thereby facilitating information transmission across time steps. Through the backpropagation through time process, RNNs can learn from past experiences and adjust their parameters to optimize their performance in sequential tasks.

**Fig. 17:**
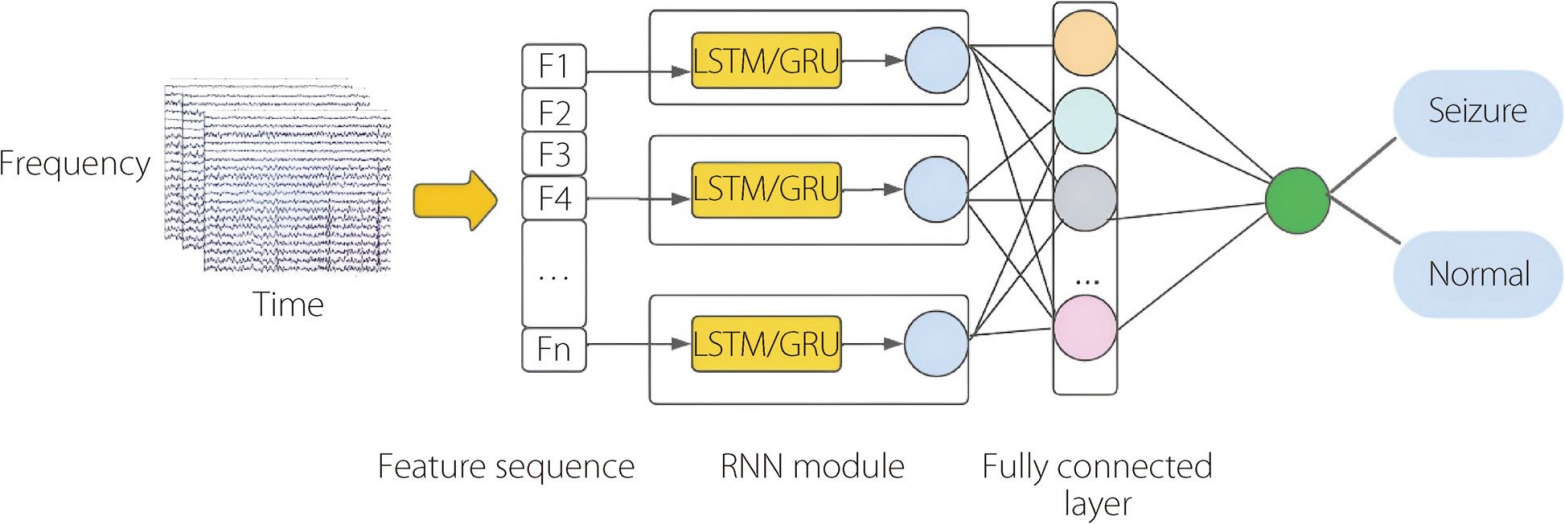
Standard workflow employing RNNs for epileptic seizure detection

**Fig. 18:**
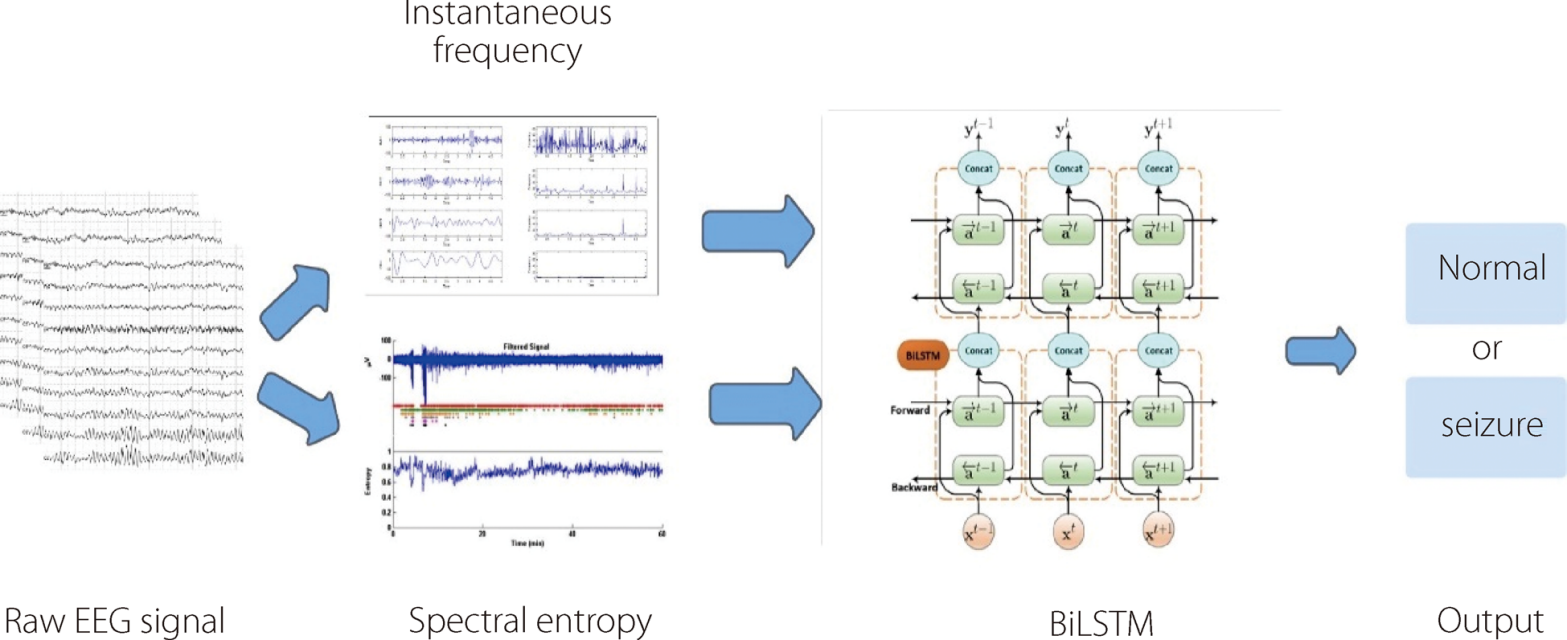
Bi-LSTM architecture for epileptic seizure detection

**Fig. 19:**
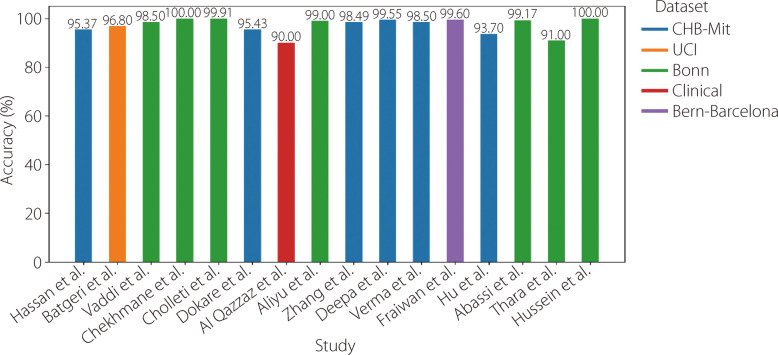
Analysis of the accuracy of results from various studies employing RNNs models for epilepsy identification

Despite their effectiveness, conventional RNNs encounter a vanishing gradient problem, which hinders their ability to capture long-term dependencies. To overcome this challenge, variants, such as LSTM and GRUs, have been designed that incorporate specialized gating mechanisms, which better preserve and update information over longer sequences. RNNs have found widespread applications across different domains, including natural language processing, speech recognition, time-series forecasting, and the generation of creative content, such as music and text. LSTM and GRUs have been extensively applied to time-series analyses, such as EEG signals. Figure 17 illustrates the standard workflow using RNNs. Tuncer and Doğru Bolat [117] used two features, instantaneous frequency and spectral entropy, with a bidirectional LSTM network to implement EEG-based epileptic seizure identification applied to the Bonn dataset. The adopted architecture, which integrates two LSTM blocks with different propagation directions to classify the brainwave signals (Fig. 18). The proposed model achieved an accuracy of 99%. Zhang et al. [118] proposed an approach that incorporates preprocessing using the WT method. The relative energies of the signals were calculated across several specific frequency bands, and the extracted features were input into a Bi-GRU network. The outputs of the Bi-GRU model were subsequently processed using MA filtering, threshold comparison, and seizure merging to determine whether the tested EEG signals indicated seizures. This approach was evaluated on the CHB-MIT dataset and achieved a sensitivity of 93.89% and a specificity of 98.49% [118]. Table 7 and Fig. 19 provide an overview of the literature on RNNs for seizure detection and prediction.

**Table 7:** Available literature using RNNs

Reference	Year	Dataset	Pre-processing	Technique	Accuracy	Sensitivity	Specificity
[119]	2025	CHB-MIT	Overlapping, filtering Normalization, PCA	LSTM	95.37%	95.80%	-
[120]	2025	UCI	Segmentation	LSTM + GRU	96.80%	96.20%	96.80%
[121]	2025	Bonn	Normalization augmentation DWT	Modified LSTM	98.50%	99.00%	98.00%
[122]	2025	Bonn	Normalization DWT	LSTM + Bi-LSTM	100.00%	100.00%	100.00%
[123]	2025	Bonn CHB-MIT	Filtering Normalization	Enhanced state refinement GRU + temporal activation regularization	99.91% 99.89%	- -	- -
[124]	2025	CHB-MIT	Normalization SMOTE Channel reduction	Spectral features + LSTM	95.43%	95.59%	95.25%
[125]	2024	Clinical	Butterworth notch filter Segmentation	Entropy-based features + LSTM + GRU + Bi-LSTM	90.00% 90.00% 90.00%	100.00% 100.00% 80.00%	80.00% 80.00% 100.00%
[126]	2023	Bonn	DWT Normalization	20 eigenvalue features with optimal CCP-selected + LSTM	99.00%	-	-
[118]	2022	CHB-MIT	DWT	Energy value + Bi-GRU	98.49%	93.89%	98.49%
[127]	2022	CHB-MIT	MinMax scaler normalization	Bi-LSTM	99.55%	-	-
[128]	2021	CHB-MIT	DWT	GRU	98.50%	-	-
[129]	2020	Bern-Barcelona	Z-score normalization Savitzky-Golay filter	Bi-LSTM	99.60%	99.55%	99.65%
[130]	2020	CHB-MIT	Segmentation local mean decomposition	Statistical features + Bi-LSTM	-	93.61%	91.85%
[131]	2019	Bonn	DCT	Hurst + ARMA features + LSTM	99.17%	98.88%	99.45%
[132]	2019	Bonn	Normalization	Stacked Bi-LSTM	91.00%	89.21%	-
[133]	2019	Bonn	Band-pass filter Segmentation	LSTM	100.00%	100.00%	100.00%

#### AEs

**Fig. 20:**
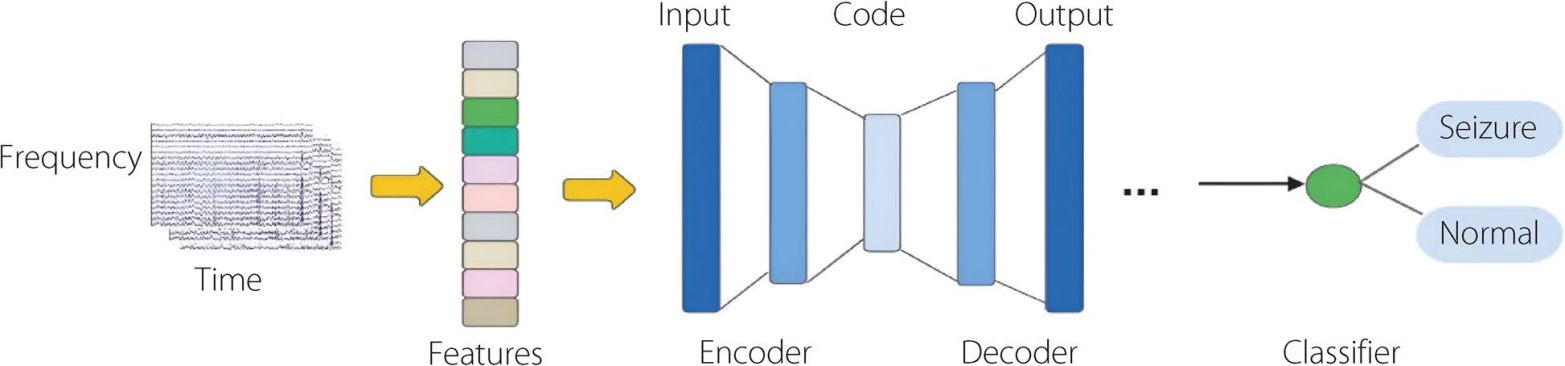
Standard workflow employing AE for epileptic seizure identification

**Fig. 21:**
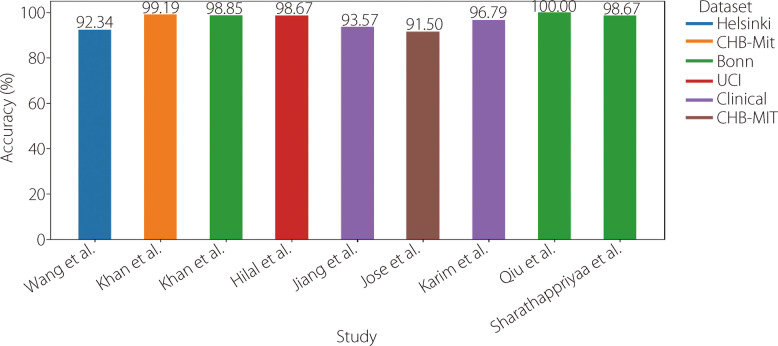
Analysis of the accuracy of results from various studies employing AEs models for epilepsy identification

AEs are NNs developed to learn compact and efficient data representations in an unsupervised manner [134]. They consist of two main components: an encoder that compresses the input and a decoder that reconstructs the original data from this representation. This enables AEs to capture the underlying data structure, which can be useful for tasks such as dimensionality reduction, detecting anomalies, and removing noise. By training on a diverse set of examples, AEs can identify key features that highlight the most relevant aspects of the data, which is invaluable for numerous ML applications. Their ability to learn from unlabeled data introduces new possibilities for understanding complex datasets. To improve the original performance, many specialized AEs have been developed, including stacked AEs, denoising AEs, and sparse AEs [135–137]. Figure 20 illustrates the common AE used for epilepsy detection and prediction [138–140]. Lin et al. [141] proposed a stacked sparse AE (SSAE) framework with a Softmax classifier for automatic epileptic seizure detection. The SSAE extracts high-level representations from the EEG data, which are then classified using a Softmax classifier. They achieved an overall average accuracy of 96% [141]. Gogna et al. [142] used a semi-supervised stacked AE to reconstruct and classify biomedical signals. Yuan et al. [140] introduced Wave2Vec, an end-to-end deep representation learning model that utilizes a CWT to extract intrinsic and temporal features before it applies sparse AEs. They achieved an accuracy of 93.92%. Table 8 and Fig. 21 present overviews of the literature on AEs for seizure detection and prediction.

**Table 8:** Available literature using AEs

Reference	Year	Dataset	Pre-processing	Technique	Accuracy	Sensitivity	Specificity
[143]	2024	Helsinki	Down sampling Filtering MinMax scaler	Time + entropy features + CAE	92.34%	98.74%	-
[144]	2023	Clinical	-	Self-attentive AE	-	74.00%	-
[145]	2023	CHBMit Bonn	Filtering Segmentation normalization	Shallow AE + SVM k-NN	99.19% 98.85%	96.10% 99.29%	99.19% 98.86%
[146]	2022	UCI	Normalization	Deep canonical sparse AE	98.67%	99.19%	99.20%
[147]	2022	CHZU	Normalization	Convolutional AE + ensemble classification model	93.57%	-	-
[148]	2021	CHB-MIT TUH	Bandpass filter	Deep stacked AE	91.50%	85.20%	86.00%
[139]	2019	Clinical	High-pass, low-pass, and notch filtering	Deep AE	96.79%	-	-
[149]	2018	Bonn	Filtering Normalization	Denoising sparse AE + LR	100.00%	100.00%	100.00%
[150]	2018	Bonn	Harmonic wavelet packet transform	Katz-based fractal dimension + AE	98.67%	-	-

#### Graph neural networks

**Fig. 22:**
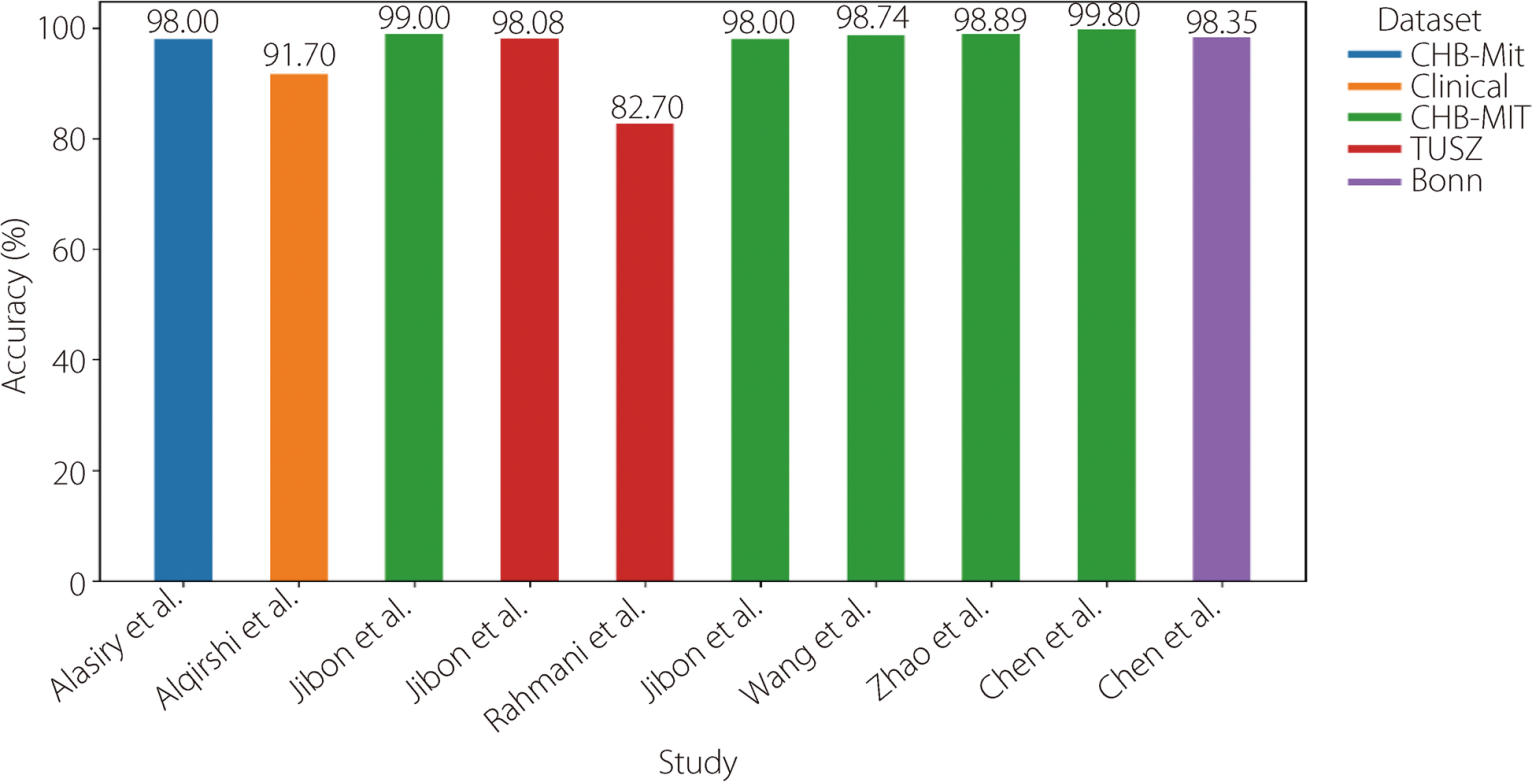
Analysis of the accuracy of results from various studies employing GNN models for epilepsy identification

Graph neural networks (GNNs) represent a specialized category of DL architectures tailored for processing data structured as graphs, where nodes signify entities and edges denote their relationships. Unlike conventional NNs, which typically operate with grid-like data, GNNs utilize the underlying graph topology to capture intricate interactions and dependencies among nodes. Through message-passing mechanisms, GNNs facilitate information exchange between neighboring nodes by iteratively refining their representations [151]. Several variants of GNNs, including graph convolutional networks (GCNs) and graph attention networks (GATs), have been employed in epilepsy detection owing to their efficacy in modeling the complex relationships and dependencies present in EEG data. EEG signals, which are inherently structured as temporal and spatial graphs, can be represented by nodes corresponding to electrodes and edges representing their connections. GNNs excel at processing this type of data by leveraging their message-passing mechanisms, thereby allowing information to be aggregated across the electrode network. This allows the capture of subtle interdependencies between different brain regions that are crucial for identifying seizure patterns. Covert et al. [152] used a temporal GCN to analyze brainwave signals using the STFT method, testing it on data from the Cleveland Clinic Foundation’s Adult Epilepsy Monitoring Unit. A linear graph convolution network (LGCN) model, proposed by Zhao et al. [153], focuses on the spatial relationships between channels. For seizure detection, the Pearson correlation matrix was used to construct an input graph, and focal loss addressed the data imbalances. Their model achieved a seizure detection accuracy score of 99.30% on the CHB-MIT dataset, outperforming existing methods without requiring manually designed features. Table 9 and Fig. 22 present overviews of the literature on GNNs for seizure detection and prediction.

**Table 9:** Available literature using GNNs

Reference	Year	Dataset	Pre-processing	Technique	Accuracy	Sensitivity	Specificity
[154]	2025	CHB-MIT	Segmentation, resampling, bandpass filter, normalization	GNN	98.00%	98.00%	-
[155]	2024	Clinical	Filtering	GCNs	91.70%	88.71%	-
[156]	2024	CHB-MIT TUH	Segmentation	SGCN-RNN	99.00% 98.08%	98.05% 95.13%	95.02% 94.99%
[157]	2023	TUSZ	FFT	Meta-learning + GNN	82.70%	-	-
[158]	2023	CHB-MIT	Stockwell Transformation	LGCN + DensNet	98.00%	-	98.60%
[159]	2022	CHB-MIT	FIR filtering Hilbert transformation	Spatiotemporal GAT	98.74%	98.87%	99.21%
[160]	2021	CHB-MIT	Band-pass filter Z-score standardization	GAT + focal loss	98.89%	97.10%	99.63%
[161]	2020	CHB-MIT Bonn	ICA	GCN	99.80% 98.35%	-	-

#### Comparative analysis of the most common neural network architectures for epilepsy detection and prediction

With the increasing complexity of EEG signal analysis, various neural network architectures have been employed to enhance classification performance. Each architecture offers unique strengths and addresses the specific challenges inherent in EEG data processing. These models have been tailored to the diversity in EEG signals by capturing spatial patterns to handle temporal dependence. Table 10 presents a comparative analysis of some of the most commonly used NN architectures, highlighting their application, advantages, and limitations in EEG signal classification. Although individual neural network architectures have demonstrated significant improvements in EEG-based epilepsy detection, limitations in capturing spatial and temporal features have driven the development of hybrid models.

**Table 10:** Comparative study of commonly used NN architectures in epilepsy detection and prediction

Neural network	Strength	Limitation	Use case in EEG
CNNs	Spatial feature extraction: CNNs are highly effective at capturing spatial patterns in EEG signals, especially when the signals are transformed into 2D representations like spectrograms, time-frequency maps, or correlation matrices Feature learning: CNNs automatically learn hierarchical features, which are useful for EEG classification tasks such as epileptic seizure detection or BCI Efficiency: With fewer parameters than fully connected networks and efficient parameter sharing, CNNs can quickly process large EEG datasets	Temporal dependencies: CNNs are not inherently designed to handle the temporal relationships present in EEG signals. They need to be paired with other models like RNNs to capture temporal dynamics	CNNs are commonly used for classifying EEG signals after transforming the raw signals into spectrograms or time-frequency maps, enabling the detection of epileptic seizures or cognitive states
RNNs	Temporal modeling: RNNs are well-suited for handling the sequential nature of EEG signals, capturing temporal dependencies and dynamic patterns over time Handling long-term dependencies: Variants like LSTM and GRU can manage long-term dependencies in EEG signals, improving the detection of abnormal brain activity that occurs across time intervals End-to-end learning: RNNs can be used directly on raw EEG data without the need for transformation into the frequency domain	Training difficulty: RNNs can be difficult to train due to vanishing gradient problems, though LSTMs and GRUs help mitigate this Slower training: RNNs especially with long sequences, can be computationally expensive and slower to train compared to CNNs	RNNs (especially LSTM/GRU) are often used for tasks requiring temporal pattern recognition in EEG signals, such as seizure prediction, sleep stage classification, or detecting cognitive states
AEs	Feature extraction: Aes are often used for unsupervised feature extraction from EEG signals. They can learn compressed, meaningful representations of the data, which are useful for classification tasks Dimensionality reduction: AEs reduce the high-dimensional EEG data into lower- dimensional latent space, which can be used as input for classifiers like SVM or neural networks Anomaly detection: Aes can detect anomalies in EEG signals by learning the typical signal structure and flagging deviations, useful in detecting abnormal brain activity such as seizures	Lack of temporal modeling: Standard aes do not capture temporal dependencies in EEG signals, though recurrent variants (e.g., RNN-based autoencoders) can handle this Supervised performance: Aes are typically used for unsupervised learning, So their performance in direct classification tasks may be less effective than supervised methods like CNNs or RNNs	Aes are used to learn meaningful features from raw EEG signals for downstream classification or anomaly detection, often in combination with other classifiers
GNNs	Capturing spatial dependencies: GNNs excel in EEG analysis when the signals are modeled as graphs (e.g., representing brain connectivity networks). They can capture the relationships between different EEG channels or regions of the brain Flexible structure: GNNs can model complex, non-Euclidean structures, making them ideal for EEG data where the connections between electrodes or brain regions carry important information Global and local information: GNNs propagate information across nodes in graph, allowing them to capture both local (e.g., within one channel) and global (across channels) dependencies	Computational complexity: GNNs can be more computationally demanding, especially when dealing with large EEG graphs or high-dimensional data Less established: Compared to CNNs and RNNs, GNNs are relatively new in EEG classification, so fewer pre-trained models and architectures are available	GNNs are used in EEG-based tasks that involve brain network analysis or connectivity, such as seizure detection based on functional or structural connectivity patterns in EEG signals

Hybrid models offer a more comprehensive approach by combining the strengths of multiple architectures and effectively leveraging the complementary capabilities of each component. The following section explores the work performed by researchers in developing these hybrid models and their impact on advancing epilepsy detection systems.

#### Hybrid DL

**Fig. 23:**
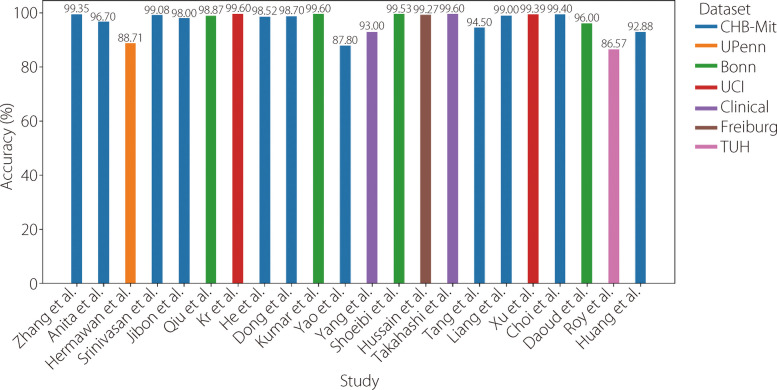
Analysis of the accuracy of results from studies employing hybrid models for epilepsy identification

Above, we reviewed previous studies on epileptic seizure detection and prediction, in which the researchers primarily focused on using individual DL models, each excelling in specific areas of capturing spatial patterns and in handling temporal dependencies in EEG data. Although these methods show promise, they often fail to fully represent the complex nature of EEG signals. Recently, researchers have begun adopting hybrid DL architectures that combine different models to benefit from their complementarity. These hybrid approaches, such as integrating CNNs for spatial features and RNNs for temporal sequences, enable more accurate and robust seizure detection and offer more effective solutions than individual techniques. Table 11 and Fig. 23 provide an overview of the literature on detection models using hybrid DL.

**Table 11:** Existing literature on the use of hybrid DL

Reference	Year	Dataset	Pre-processing	Hybrid deep neural network	Hybrid deep neural network detail	Accuracy	Sensitivity	Specificity
[162]	2025	SWEC-ETHZ HUP	Notch filter normalization	CNN + Transformer	3Conv + 3LN + 3Relu + 2 Transformer Encoder	91.15% 88.84%	89.48% 87.41%	92.82% 90.27%
[163]	2025	CHB-MIT Bonn	Segmentation	Transformer + CNN + ResNet + ViT	Transformer Encoder + 2CNN + ResNet-18 + ViT + Fusion layer + MLP	98.70% 99.70%	98.30% 98.90%	99.00% 99.40%
[164]	2025	Bonn CHB-MIT	Butterworth filter DWT	CNN + Bi-LSTM	2Conv + 1 Max Pooling + 1 Bi-LSTM + 1 FC + Softmax	96.19% 98.43%	95.08% 97.84%	97.34% 99.21%
[165]	2025	Bonn	Butterworth filter Normalization	CNN + ResBiGRU + CBAM	1Conv + 1BN + 1MP + 1 Dropout + 1ResBiGRU + 1 CBAM + Dense + Softmax	99.00%	99.00%	99.00%
[166]	2025	CHB-MIT	Notch filter Segmentation	EfficientNet-B0 + SVM	-	96.12%	95.21%	-
[167]	2025	Bonn	Adaptive stationary orthonormal WTs	ResNet + Bi-LSTM + Attention	7Conv + 1BN + 1Relu + 1MP + 1Bi-LSTM + 2FC + Softmax	94.00%	93.00%	87.70%
[168]	2025	CHB-MIT SH-SDU	Segmentation DWT	CNN + Informer	3Conv + 3BN + Elu + 2 Max Pool + 1 Dropout + 3 Informer Encoders + 3 FC	98.54% 92.78%	99.54% 95.40%	98.55% 92.92%
[169]	2025	TUSZ	Segmentation, FFT normalization augmentation	LSTM + GAT	1 LSTM + Gaussian connection module + GAT + 1 FC + Softmax	91.00%	-	-
[170]	2025	Kaggle	Normalization segmentation	Hybrid attention + Transformer + CNN	1Conv + Self-attention + Cross-attention + Transformer Encoder + 1FC	98.30%	95.90%	98.21%
[171]	2024	CHB-MIT Siena	Segmentation Stockwell transform	GANs + Bi-LSTM	-	- -	92.55% 83.53%	92.18% 81.88%
[172]	2024	CHB-MIT	DWT	CNN-GRU-Atten	2Conv + 1GRU + 1Atten + 1FC	99.35%	99.24%	99.51%
[173]	2024	CHB-MIT	Butterworth filter Normalization	CNN-Bi-LSTM Atten	3Conv + 2Bi-LSTM + 1Atten + 1FC + Softmax	91.94%	-	93.00%
[174]	2024	CHB-MIT Siena Helsinki	Filtering Normalization	CNN-Bi-LSTM	8Conv + 2Bi-LSTM + 1Dropout + MLP	92.80%	95.00%	90.30%
[175]	2024	CHB-MIT	FBSE-EWT	MSA-DCNN-LSTM	5Conv + LSTM + Dense	96.70%	96.70%	96.70%
[176]	2024	UPenn Melbourne	Butterworth filter L2 normalization	CNN-LSTM	1Conv + 2LSTM + Dropout + 2Dense	88.71% 81.33%	- -	- -
[177]	2023	CHB-MIT	Normalization	3D-DCAE-Bi-LSTM	4Conv + 1Bi-LSTM + 1Pooling	99.08%	99.21%	99.11%
[178]	2023	Bonn Hauz Khas	-	ResNet-LSTM-Atten	ResNet + LSTM + 4Atten + 2FC	98.87%	-	-
[179]	2023	CHB-MIT Bonn Kaggle	Filtering downsampling Segmentation	CNN-Bi-GRU-Atten	3Conv + 1Bi-GRU + Cross-Layer-Atten + Pool + Softmax	98.50%	98.50%	98.80%
[180]	2023	UCI	Normalization	CNN-Bi-LSTM	2Conv + 1Bi-LSTM + 4FC + Softmax	99.60%	-	99.30%
[181]	2022	CHB-MIT TUH	Bandpass filter Normalization	GAT-Bi-LSTM	2GAT + 1Bi-LSTM + 1FC + Softmax	98.52% 98.02%	97.75% 97.70%	94.34% 99.06%
[182]	2022	CHB-MIT	Bandpass filter Normalization	Atten-based graph ResNet with focal loss	-	98.70%	97.94%	98.66%
[183]	2022	Bonn	Bandpass filter	2D-CNN-GRU	-	99.60%	-	-
[184]	2021	CHB-MIT	-	Atten-Bi-LSTM	1Bi-LSTM + 1Atten + Time Distributed FC + GAPooling + FC + Softmax	87.80%	87.30%	88.30%
[185]	2021	Clinical	-	CNN-LSTM	3Con + 1LSTM + 3BN + 2Pooling + 4Dropout + 3FC + Sigmoid	-	88.00%	92.00%
[186]	2021	Bonn	Time, statistical, non-linear features fisher feature scoring algorithm	CNN-AE	5Conv + 4Pooling + 3BN + Dropout + Softmax	99.53%	-	-
[187]	2021	Freiburg	FTT DWT	1DCNN-LSTM	1DConv + 1LSTM + Max Pooling + 1FC + Softmax	99.27%	-	-
[188]	2021	Bonn CHB-MIT	Bandpass filter segmentation	MVKRVFLN-DLSTM	3LSTM + MVKRVFLN classifier	100.00% 99.97%	100.00% 100.00%	100.00% 99.98%
[189]	2020	Clinical	Filtering	CNN-AE	1AE + VGG16	99.60%	-	-
[190]	2020	CHB-MIT	Decomposition multi-view feature extraction	Multi-view CNN-GRU	9Conv + 9BN + 6Pooling + Relu + 1GRU + 1Atten + 1FC + Softmax	-	94.50%	-
[191]	2020	TUSZ	DA	2DCNN-LSTM	6Conv + 6Pooling + 6BN + Relu + LSTM + FC + Dropout + Softmax	-	86.00%	-
[192]	2020	CHB-MIT	Conversion into a series of two seconds waveform images	LRCN	10Conv + 5Pooling + 1LSTM + Softmax	99.00%	84.00%	99.00%
[193]	2020	UCI	Normalization	1DCNN-LSTM	4Conv + Pooling + 2LSTM + Dropout + 3FC	99.39%	-	-
[194]	2019	CHB-MIT SNUH	STFT 2D mapping	3DCNN-Bi-GRU	-	99.40%	98.00%	99.50%
[195]	2019	CHB-MIT	STFT	CNN-AE	16Conv + 15Pooling + Softmax	96.22%	-	-
[196]	2019	Bonn	DA	CDAE	Encoder: 2Conv + 2Pooling + 2BN Decoder: 2de-Conv + 2Upsampling + 2BN + MLP	96.00%	93.00%	99.00%
[197]	2019	CHB-MIT	Segmentation	Atten-CNN-Bi-RNN	4Conv + 1Atten + 2Bi- LSTM + 1FC + Softmax	-	92.88%	93.94%
[198]	2019	TUH	Data selection	1DCNN-GRU (ChronoNet)	9Conv + 4GRU + Softmax	86.57%	-	-

## Discussion and challenges

The application of ML and DL techniques for detecting and predicting epilepsy has gained significant traction, highlighting their potential to transform clinical practice. This study represents a thorough systematic analysis of methodologies based on traditional ML and DL technologies to explore the complexities of frequently used NN architectures in detail. First, the essential steps involved in the pre-processing and feature extraction methods specific to neuroimaging modalities were meticulously examined, with particular emphasis on EEG signals. The significance of these initial stages cannot be overstated, as they lay the groundwork for subsequent analysis and classification tasks. Second, various studies were explored that employ conventional ML methods found in the field of seizure detection and prediction, demonstrating notable efficacy despite inherent limitations in their capability to handle complex, high-dimensional data. As shown in Table 3, high-performing studies frequently utilized datasets such as Bonn, CHB-MIT, and EPILEPSIAE, coupled with extensive preprocessing techniques such as DWT, statistical feature extraction, PCA, and artifact removal. For example, Ikizler and Ekim [62] achieved perfect classification accuracy (100%) using the Bonn dataset with PSD features and classifiers like RF, k-NN, and MLP. Similarly, Liu et al. [70] combined filtering and parametric PSD on the Bonn and CHB-MIT data to reach accuracies up to 99.95%. The use of TreeBagger in ref. [64], applied to clinical EEG data, demonstrated strong generalizability with an accuracy of 91.5% and a sensitivity of 97%, outperforming simpler models like DT and LR in the same study. Ensemble methods, such as RF, AdaBoost, and Gradient Boosting in ref. [66], also showed competitive results across multiple datasets, with extra trees achieving a balanced performance of 94.3% in accuracy, sensitivity, and specificity. DL approaches with CNN-based artifact removal ( [65, 67]) have exhibited promising outcomes, particularly in reducing false-positive rates, although traditional models still trail in sensitivity. Pattnaik et al. [73] and Harpale and Bairagi [74] emphasized hybrid preprocessing (e.g., tunable Q-WT, ICA) combined with SVM and fuzzy classifiers, consistently achieving > 90% accuracy. However, some methods, such as EMD-DWT in ref. [69], underperform with accuracies of < 60%, highlighting the critical role of appropriate feature engineering. Overall, models integrating advanced signal decomposition, ensemble learning, and rigorous preprocessing tend to yield superior results to certain classical models, such as SVM, which continue to offer strong performance when paired with optimized features. Third, recent advancements in DL approaches that have revolutionized the landscape of seizure detection were comprehensively reviewed. These DL models leverage their ability to extract hierarchical features automatically from raw EEG data, offering superior performance and robustness compared with traditional methods. A comparative analysis of recent studies on epileptic seizure prediction highlights the distinct advantages and capabilities of CNN-, RNN-, AE-, and GNN-based models. CNN-based approaches have demonstrated consistently high performance, particularly with time-frequency representations like CWT, STFT, and spectrograms, achieving accuracy rates up to 100% in several studies ( [108, 110]). These models benefit from their ability to extract robust spatial features from EEG signals. In contrast, RNN variants, particularly LSTM and Bi-LSTM networks, excel at modeling temporal dependencies, with multiple studies reporting perfect accuracy and sensitivity ( [112, 133]). Moreover, hybrid RNN architectures, combining GRU or attention mechanisms, further enhance performance on both clinical and public datasets. AE-based methods, including convolutional and sparse variants, are increasingly adopted for unsupervised feature extraction, achieving competitive results (e.g., 100% accuracy in ref. [149]) while enabling dimensionality reduction and denoising. However, their performance can vary depending on the architecture depth and classifier integration. GNN models, such as GCNs and attention-based networks, offer promising alternatives by leveraging the spatial relationships between EEG channels. Recent studies have reported accuracies of up to 99.8% ( [161]) and strong generalization for clinical and large-scale datasets. Overall, while CNNs and RNNs remain dominant regarding predictive accuracy, AEs offer interpretability and compression benefits, while GNNs provide a powerful framework for capturing spatiotemporal dependencies, well-suiting them for complex, multi-channel EEG data. Although standalone DL models have demonstrated strong performance in epilepsy detection and prediction, their individual limitations, such as the CNNs struggling with temporal dependencies and RNNs facing computational inefficiencies, have led researchers to explore hybrid architectures. Recently, studies have increasingly focused on combining the strengths of various types of NNs. A comparative analysis of recent state-of-the-art works revealed that hybrid DL architectures have emerged as powerful solutions to the standalone models’ limitations in epileptic seizure detection. Most notably, CNNs combined with temporal models, such as LSTM, Bi-LSTM, GRU, and Transformer encoders, consistently outperform single-network approaches by capturing both the spatial and temporal dependencies inherent in EEG signals. For instance, the integration of CNNs with Bi-LSTM and attention mechanisms ( [164, 173, 174]) has achieved high accuracy, balanced sensitivity, and specificity, highlighting the benefits of sequential modeling in capturing complex seizure patterns. Similarly, architectures incorporating advanced attention mechanisms or graph-based networks ( [167, 169, 181, 182]) have demonstrated superior performance in addressing inter-subject variability and enhancing feature representation. Multibranch or fused models, such as those combining ResNet, ViT, and Transformer modules ( [163, 170]), further boost classification performance by integrating global and local feature extraction. However, these gains are accompanied by increased complexity and computational demands, emphasizing the need for trade-offs between accuracy and efficiency.

This comprehensive analysis of existing state-of-the-art literature shares several key lessons. A primary insight is that the noise and complexity of EEG signals demand preprocessing and feature extraction. EEG data are often contaminated with artifacts and exhibit nonstationary low-amplitude characteristics, making direct analysis challenging. Effective preprocessing techniques, such as filtering and artifact removal, are essential for enhancing signal quality and reliability. Likewise, robust feature extraction methods are vital for capturing meaningful patterns embedded in both the temporal and spectral domains.

Recent studies have demonstrated that well-designed preprocessing pipelines and domain-specific features can significantly improve model performance and accuracy. In Addition, hybrid DL architectures, particularly those combining convolutional, recurrent, and attention mechanisms, have pertinently demonstrated superior performance. As such, future work should focus on optimizing these hybrid designs for generalization across datasets while maintaining the computational feasibility for real-time applications. The progression from conventional ML to DL signifies a pivotal shift in the approach to seizure detection, which acknowledges the complexity and dynamics of brainwave signals. Traditional methods rely heavily on handcrafted features and rule-based systems, which often fail to capture EEG signals’ complex nonlinear characteristics. Conversely, recent advancements in ML algorithms, particularly DL models such as CNNs, RNNs, AEs, and GNNs, have demonstrated remarkable performance by automatically learning relevant features directly from data. Furthermore, hybrid models that integrate multiple architectures have emerged, effectively combining the strengths of different approaches to enhance detection accuracy and robustness. Despite these advancements, the following challenges remain:

### Data reliability and disparity

Ensuring the acquisition and preparation of high-quality EEG signals for training ML and DL architectures remains a challenge. Additionally, class imbalance is a concern because seizure episodes occur less frequently than non-seizure periods. To enhance the variety and volume of the training data, augmentation methods, including SMOTE and generative adversarial networks, can be employed to create artificial data. Approaches such as class weighting and resampling can be used to address the challenges posed by class imbalances. Furthermore, fostering collaboration among research institutions is essential for collecting EEG recordings that are accessible to the broader scientific community, ultimately enhancing research output quality and accuracy.

### Interpretability

The interpretability of the DL architecture is a significant concern. Although CNN and RNN architectures are highly efficient, they are typically viewed as “black-box” models, with little understanding of how they extract features and their reasoning. Improving the interpretability and trustworthiness of these models is an important research area. Techniques such as Grad-CAM, SHAP, and layer-wise relevance propagation can provide insight into such decision-making processes. In Addition, employing less complex or hybrid architectures that integrate DL with more transparent models can help achieve an equilibrium between efficiency and interpretability.

### Restricted computing resources

The implementation of DL architectures in real-world applications poses significant issues concerning computational capabilities and model refinement. Computational burdens can be alleviated by prioritizing the creation of lightweight architectures. Furthermore, cloud-based solutions and edge computing may provide viable pathways for deploying such architectures in environments with limited resources.

Future studies should focus on addressing these challenges and exploring the integration of real-time monitoring systems to facilitate timely interventions and improve patient lives.

## Conclusions

This review comprehensively examined the latest advancements in the detection and prediction of epileptic seizures. It analyzes various methods, starting with traditional signal processing techniques as a fundamental step. Several solutions proposed by researchers employing ML approaches and the latest DL algorithms were also investigated. The strengths and weaknesses of each technique were highlighted, emphasizing the importance of addressing challenges, such as the dynamic nature of EEG signals, low signal-to-noise ratios, and inter-subject diversity.

This study emphasizes the need for robust and generalizable models that can effectively handle the diverse and dynamic characteristics of EEG data. Future research should prioritize the development of adaptive algorithms and, over time, improve their ability to learn from continuous data streams. Additionally, there is a pressing need for large standardized datasets to enable more comprehensive model training and evaluation.

A limitation of this review is the scarcity of studies using a single DL model for seizure detection since most research focuses on hybrid architectures that combine several techniques to improve performance. Although they have yielded promising results, these models tend to be more complex and less interpretable. Furthermore, the lack of publicly available multimodal datasets, such as those combining EEG with MRI or PET scans restricts the exploration of complementary information that could potentially enhance the accuracy and robustness of seizure detection models. Future research would benefit from publicly available multimodal datasets and studies exploring the use of individual DL models.

The integration of multimodal data and advanced techniques such as transfer learning and federated learning holds promise for improving the performance and reliability of seizure detection and prediction systems. By addressing these challenges, the field can move closer to achieving accurate real-time monitoring and interventions, ultimately reducing the burden of epilepsy and improving the quality of life.

## Data Availability

All publicly available datasets used in the studies reviewed in this review are described in this section. CHB-MIT dataset is available at https://physionet.org/content/chbmit/1.0.0/. Bonn dataset is available at https://www.ukbonn.de/epileptologie/arbeitsgruppen/ag-lehnertz-neurophysik/downloads/. Freiburg dataset is available at https://www.epilepsy.uni-freiburg.de/freiburg-seizure-prediction-project/EEG-database. Kaggle competition on seizure prediction dataset (UPenn and Mayo Clinic’s Seizure Detection Challenge) is available at https://www.kaggle.com/c/seizure-detection/data. TUH Corpus (TUSZ) is available at https://isip.piconepress.com/projects/nedc/html/tuh_eeg/. Hauz Khas dataset is available at https://www.researchgate.net/publication/308719109_EEG_Epilepsy_Datasets. Melbourne dataset is available at https://figshare.unimelb.edu.au/articles/dataset/Seizure_Data/6939809. Siena dataset is available at https://physionet.org/content/siena-scalp-eeg/1.0.0/. Helsinki dataset is available at https://zenodo.org/records/2547147#.Y7eU5uxBwlI.

## Abbreviations

EEG: Electroencephalography
AI: Artificial intelligence
MEG: Magnetoencephalography
fMRI: Functional magnetic resonance imaging
PET: Positron emission tomography
ML: Machine learning
DWT: Discrete wavelet transform
EMD: Empirical mode decomposition
RNN: Recurrent neural network
GRU: Gated recurrent unit
CNN: Convolutional neural network
LSTM: Long short-term memory
DL: Deep learning
BCI: Brain-computer interface
CHB-MIT: Children’s Hospital Boston-Massachusetts Institute of Technology
ICA: Independent component analysis
IMF: Intrinsic mode function
PCA: Principal component analysis
WPD: Wavelet packet decomposition
WT: Wavelet transform
FFT: Fast fourier transform
AR: Autoregressive
ARMA: Autoregressive moving average
MA: Moving average
CWT: Continuous wavelet transform
SVM: Support vector machine
RF: Random forest
PSD: Power spectral density
STFT: Short-time Fourier transform
RQA: Recurrence quantification analysis
LDA: Linear discriminant analysis
ANN: Artificial neural network
LR: Logistic regression
AE: Autoencoder
PHK: Prince Hospital Khulna
LGCN: Linear graph convolution network
k-NN: K-nearest neighbors
MLP: Multilayer perceptron
SSAE: Stacked sparse autoencoder
GNN: Graph neural network
GCN: Graph convolutional network
GAT: Graph attention network
